# A community-based lifestyle and weight loss intervention promoting a Mediterranean-style diet pattern evaluated in the stroke belt of North Carolina: the Heart Healthy Lenoir Project

**DOI:** 10.1186/s12889-016-3370-9

**Published:** 2016-08-05

**Authors:** Thomas C. Keyserling, Carmen D. Samuel-Hodge, Stephanie Jilcott Pitts, Beverly A. Garcia, Larry F. Johnston, Ziya Gizlice, Cassandra L. Miller, Danielle F. Braxton, Kelly R. Evenson, Janice C. Smith, Gwen B. Davis, Emmanuelle L. Quenum, Nadya T. Majette Elliott, Myron D. Gross, Katrina E. Donahue, Jacqueline R. Halladay, Alice S. Ammerman

**Affiliations:** 1 Division of General Medicine and Clinical Epidemiology, Department of Medicine, School of Medicine, CB 7110, University of North Carolina, 5039 Old Clinic Building, Chapel Hill, NC 27599 USA; 2 Center for Health Promotion and Disease Prevention (a CDC Prevention Research Center), CB 7426, University of North Carolina, Chapel Hill, NC 27599 USA; 3 Department of Public Health, Brody School of Medicine, East Carolina University, Lakeside Annex 8, 600 Moye Blvd, MS 660, Greenville, NC 27834 USA; 4 Department of Nutrition, Gillings School of Global Public Health, CB 7461, University of North Carolina, Chapel Hill, NC 27599 USA; 5 Department of Epidemiology, Gillings School of Global Public Health, CB 8050, University of North Carolina, Chapel Hill, NC 27599 USA; 6 Greene County Health Department, 225 Kingold Blvd, Suite B, Snow Hill, North Carolina 28580 USA; 7 Student Health Services, East Carolina University, 1000 East 5th St, MS 408, Greenville, NC 27858 USA; 8 Department of Laboratory Medicine and Pathology, University of Minnesota, Minneapolis, MN 55455 USA; 9 Department of Family Medicine, School of Medicine, CB 7595, University of North Carolina, Chapel Hill, NC 27599 USA; 10 Cecil G. Sheps Center for Health Services Research, School of Medicine, CB 7590, University of North Carolina, Chapel Hill, NC 27599 USA

**Keywords:** Mediterranean diet, Dietary intervention, Lifestyle intervention, Weight loss intervention, Low-income participants, Disparities, Cardiovascular disease, Prevention

## Abstract

**Background:**

Because residents of the southeastern United States experience disproportionally high rates of cardiovascular disease (CVD), it is important to develop effective lifestyle interventions for this population.

**Methods:**

The primary objective was to develop and evaluate a dietary, physical activity (PA) and weight loss intervention for residents of the southeastern US. The intervention, given in eastern North Carolina, was evaluated in a 2 year prospective cohort study with an embedded randomized controlled trial (RCT) of a weight loss maintenance intervention. The intervention included: Phase I (months 1–6), individually-tailored intervention promoting a Mediterranean-style dietary pattern and increased walking; Phase II (months 7–12), option of a 16-week weight loss intervention for those with BMI ≥ 25 kg/m^2^ offered in 2 formats (16 weekly group sessions or 5 group sessions and 10 phone calls) or a lifestyle maintenance intervention; and Phase III (months 13–24), weight loss maintenance RCT for those losing ≥ 8 lb with all other participants receiving a lifestyle maintenance intervention. Change in diet and PA behaviors, CVD risk factors, and weight were assessed at 6, 12, and 24 month follow-up.

**Results:**

Baseline characteristics (*N* = 339) were: 260 (77 %) females, 219 (65 %) African Americans, mean age 56 years, and mean body mass index 36 kg/m^2^. In Phase I, among 251 (74 %) that returned for 6 month follow-up, there were substantial improvements in diet score (4.3 units [95 % CI 3.7 to 5.0]), walking (64 min/week [19 to 109]), and systolic blood pressure (−6.4 mmHg [−8.7 to −4.1]) that were generally maintained through 24 month follow-up. In Phase II, 138 (57 group only, 81 group/phone) chose the weight loss intervention and at 12 months, weight change was: −3.1 kg (−4.9 to −1.3) for group (*N* = 50) and −2.1 kg (−3.2 to −1.0) for group/phone combination (*N* = 75). In Phase III, 27 participants took part in the RCT. At 24 months, weight loss was −2.1 kg (−4.3 to 0.0) for group (*N* = 51) and −1.1 kg (−2.7 to 0.4) for combination (*N* = 72). Outcomes for African American and whites were similar.

**Conclusions:**

The intervention yielded substantial improvement in diet, PA, and blood pressure, but weight loss was modest.

**Trial registration:**

clinicaltrials.gov Identifier: NCT01433484

**Electronic supplementary material:**

The online version of this article (doi:10.1186/s12889-016-3370-9) contains supplementary material, which is available to authorized users.

## Background

In the United States (US), large disparities in cardiovascular disease (CVD) exist among subgroups defined by race, ethnicity, socioeconomic status (SES), and geography (with rates high in the Southeast, particularly in the “stroke belt”) [1–6]. The reasons are complex and include differences in genetic factors, access to and quality of care [7, 8], and lifestyle factors [5, 6]. Notably, individuals of low SES and minority status, and more generally, residents of the southeastern US, typically consume less fruit and vegetables [9–12], engage in less leisure-time physical activity (PA) [13, 14], and are more likely to be obese [15] compared to higher SES, non-minority, and non-southeastern counterparts. As diet and PA behaviors are modifiable and, if sustained, are associated with substantial reduction in CVD risk [16, 17], improving diet and PA behaviors in high risk groups affords an opportunity to substantially reduce CVD disparities.

Dietary patterns that include frequent consumption of high quality fats (polyunsaturated and monounsaturated fats primarily from plant sources and fish) and carbohydrates (fruits, vegetables, and whole grains) are associated with large reductions in CVD risk [16, 18, 19]. In the PREDIMED randomized trial [20], a Mediterranean dietary pattern supplemented with nuts or olive oil reduced CVD risk by 30 % in the intervention groups. While the Mediterranean dietary pattern has been well studied in Europe [21–23] and in selected US populations [24–28], it has not been evaluated in high risk, largely minority populations in the southeastern US. Further, the Mediterranean dietary pattern holds promise for weight loss interventions as it may be easier to maintain over time, especially compared to low fat diets [29].

Given the importance of dietary patterns in reducing CVD risk [30], and the burden of CVD risk among residents of the “stroke belt” in eastern North Carolina [4], we developed and evaluated a culturally appropriate lifestyle intervention for this population, with a major focus on improving diet quality while also promoting PA and weight loss. In this paper we report the intervention’s effect on diet and PA behaviors, CVD risk factors, and weight loss through 24 months of follow-up. Because a major focus of this research was to reduce disparities in CVD risk, outcomes are also reported by race, as African Americans are at increased risk for CVD [3], but often have poorer outcomes compared to whites in response to lifestyle and weight loss interventions [31–34].

## Methods

### Study overview

Situated in eastern North Carolina, Lenoir County is located in the “stroke belt,” [4] with elevated heart disease and stroke rates relative to other regions of North Carolina [35] and national levels [36]. This study was part of the Heart Healthy Lenoir (HHL) Project [37], a collaborative research effort designed to reduce CVD risk and disparities in risk in Lenoir County. This paper reports on the lifestyle intervention study, one of 3 coordinated HHL studies which also included a study to improve high blood pressure (BP) management at local practices and a study examining associations between genetic markers and change in CVD risk factors. Lifestyle study participants were recruited from the community and also included some participants who took part in the HHL high BP study [38]. The study was designed and conducted with input from a local Community Advisory Committee (CAC) [37] with data collected from September 20, 2011 to November 7, 2014 and analyzed in 2015 and 2016.

**Fig. 1:**
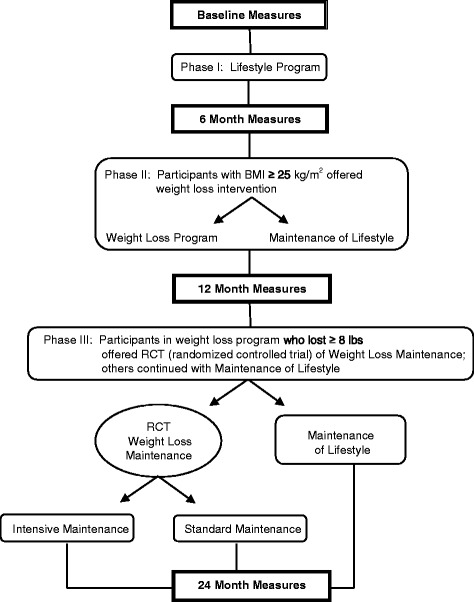
Study overview

The lifestyle study included 3 phases, as depicted in Fig. 1. Phase I, lasting 6 months and given to all participants, focused on improving diet quality and increasing PA. In Phase II, also 6 months in length, participants with a body mass index (BMI) ≥ 25 kg/m^2^ were invited to take part in a weight loss intervention. Those who did not and those with a BMI < 25 kg/m^2^ received a lifestyle maintenance intervention. Phase III, lasting a year, included a randomized controlled trial (RCT) comparing a more intensive to less intensive weight loss maintenance intervention for those who lost ≥ 8 lb in Phase II and a lifestyle maintenance intervention for the other participants. We did not include a control group in Phases I and II because we had previously shown lifestyle and weight loss interventions given in similar formats to low SES participants were effective when evaluated in randomized trials [39–42] and we wanted to offer a lifestyle and weight loss interventions to as many community members as possible. Additionally, our CAC strongly encouraged a study design in which all participants received “active treatment.”

### Participants

The enrollment goal for the lifestyle study was 350 participants; approximately 150 enrolled from the community and 200 from the high BP study. The rational for including BP study participants was their increased risk for CVD and likelihood of benefit from receiving the lifestyle intervention. To recruit from the community, the study was publicized through flyers, newspaper articles, television notices, word of mouth, and the study website. In an effort to enroll a representative community sample, the only inclusion criteria for screening were age ≥ 18 years and interest in improving diet and PA behaviors to reduce CVD risk. Screening criteria for the high BP study were age ≥ 18, established patient at a participating practice, and a systolic BP ≥ 150 mmHg when assessed during routine care within the past 12 months. Participants who attended the enrollment visit for the high BP study were invited to take part in the lifestyle study until 200 agreed to do so.

Research staff screened potential participants (primarily by phone) to determine if they met the following additional eligibility criteria: lived in or near Lenoir County, North Carolina; spoke English; had access to a telephone; no drug or alcohol abuse within the past two years; did not have advanced kidney disease or dementia; did not have psychosis; and did not have a history of malignancy, other than non-melanoma skin cancer, unless surgically cured > 5 years ago or in remission. If so, they were invited to the enrollment visit, conducted at a central research office or the participants’ clinics. To identify potential safety issues related to increasing PA, a modified version of the Physical Activity Readiness Questionnaire [43] was given. Those who screened positive or had a myocardial infarction during the preceeding 3 months were required to get approval from their physician to take part in the PA component of the intervention.

### Lifestyle study intervention

#### Phase I (months 1–6) diet and PA intervention

The lifestyle study intervention focused on dietary and PA behaviors and did not address other aspects of lifestyle relevant to CVD risk reduction, such as smoking cessation. The intervention framework was originally developed by Ammerman and colleagues [44, 45], tested in a variety of clinical settings in North Carolina [46–48], and was previously updated to include a greater focus on carbohydrate quality [39]. To be consistent with the evolving literature on the importance of high quality fats in reducing CVD risk [49–56], the intervention was further revised for this study to include a major focus on improving fat quality. With this modification, the intervention dietary pattern closely resembled that tested in the nut intervention arm of the PREDIMED study; thus, we call it the “Med-South Diet,” given the focus on the Southeastern US. As shown in Table 1, 9 of the 13 major recommendations advocated by the PREDIMED intervention diet [20] were almost identical to those in the Med-South Diet.

**Table 1:** PREDIMED Diet goals^20^ compared to Med-South Diet goals^a^

Food	PREDIMED Diet goal	Med-South Diet goal
1--Vegetable Oil	Olive oil group: ≥ 4 tbsp/day extra virgin olive oil	2–6 servings/day of foods high in healthy fats (nuts, fish, full fat salad dressings and spreads, other foods prepared with olive or vegetable oils, and vegetables with high fat content such as avocado)
*2--Tree nuts and peanuts*	*Nut group: ≥ 3 servings/wk* ^*b*^	*≥3 servings/wk*
*3--Fresh fruits*	*≥3 servings/day*	*≥7 servings for fruits and vegetables/day*
*4--Vegetables*	*≥3 servings/day*	
5--Fish (especially fatty fish), seafood	≥3 servings/wk	≥1 servings/wk
*6--Legumes*	*≥3 servings/wk*	*≥3 servings/wk*
7--Sofrito^b^	≥2 servings/wk	No recommendation
*8--White meat*	*Instead of red meat*	*Consume poultry often*
*9--Wine with meals (optional, only for habitual drinkers)*	*≥7 glasses/wk*	*Do not recommend starting wine consumption, but provide information on effects of alcohol for heart health, suggesting up to 1 serving a day for females and up to 2 for males*
*10--Soda drinks*	*<1 drink/day*	*<1 drink/day*
*11--Commercial bakery goods, sweets, and pastries*	*<3 servings/wk*	*<3 servings/wk*
12--Spread fats	<1 serving/day	Up to several servings/day of *trans* fat free spreads
*13--Red and processed meats*	*<1 serving/day*	*≤1 serving/day*

*Abbreviations: PREDIMED* Prevención con Dieta Mediterránea, *tbsp* tablespoon, *wk* week

^a^Rows in italics indicate identical or nearly identical dietary recommendations

^b^The PREDIMED intervention recommended nuts daily, but considered nut consumption ≥ 3 servings/wk to be adherent to the nut recommendation

^c^Sofrito is a sauce made with tomato and onion, often including garlic and aromatic herbs, and slowly simmered with olive oil

The Phase I intervention, described in detail in Section 1a of Additional file 1, included 4 monthly sessions delivered by a trained counselor. Dietary counseling comprised about ¾ of intervention content and time; the rest was devoted to PA counseling, with a goal of walking ≥ 7,500 steps/day or ≥ 30 min on at least 5 days/week. Participants could choose the intervention format -- either 60-min individual counseling sessions or 120-min group sessions [57, 58] -- which were given at a central research office or the participants’ clinics for those also enrolled in the high BP study. Spouses and friends were invited to intervention sessions and for those who could not attend in person, counseling was offered by telephone. Participants also received an illustrated guide listing local community resources for healthy eating and PA and a pedometer and activity logs for self-monitoring PA.

At the first counseling session, the lifestyle survey (Section 1b of Additional file 1) was administered, consisting of the Dietary Risk Assessment (DRA), describe in detail below and 11 items assessing the amount and intensity of PA. Then, an overview of the program was given and the specific dietary content of Session 1 was reviewed. At the end of the session, the participant and counselor developed an individually tailored action plan to help guide the participant’s eating behaviors for the next month (or until the next counseling session). To do so, they first reviewed the lifestyle survey page for Session 1 (Section 1b of Additional file 1, page A-3) and identified current eating behaviors that “could be improved” or “need to be improved.” Then they reviewed the dietary tips for these problematic eating behaviors on the tip sheet for Session 1 (Section 1c of Additional file 1, page B-24), with tips linked by number and color coding to the items on the lifestyle survey. Of note, the tip sheet included recipe suggestions in a “Southern Style” cookbook given to all participants. Finally, the counselor and participant identified 2 achievable goals (participants could opt to choose 1) to work on before the next visit and documented them on the goal sheet (Section 1d of Additional file 1, page B-28). If time permitted during Session 1, PA was also addressed in a similarly tailored way but with only one goal selected. Subsequent sessions followed the same format but opened with a check-in on progress toward goals and addressed diet and PA.

#### Phase II (months 7–12)--weight loss and lifestyle maintenance intervention

Participants with a BMI ≥ 25 kg/m^2^ could elect to take part in the weight loss intervention; those who did not and those with a BMI of < 25 kg/m^2^ received a lifestyle maintenance intervention consisting of 3 phone calls, as previously described [59]. The weight loss intervention was offered in 2 formats over approximately 16 weeks: weekly group sessions (16) as previously tested [40, 41, 60], or 5 group sessions plus 10 phone contacts (combination intervention) as outlined in Section 2 of Additional file 1. The major modification from the previously tested weight loss intervention was the focus on the Med-South dietary pattern and addition of newer evidence-based behavioral components (e.g., daily self-weighing) [61].

#### Phase III (months 13–24)-- weight loss and lifestyle maintenance interventions

Participants losing ≥ 8 lb could elect to take part in the weight loss maintenance RCT; those who did not and all other study participants received brief, quarterly lifestyle maintenance intervention phone calls. The first 2 calls addressed diet and PA and, to tailor counseling, began with the counselor administering a subset of items from the lifestyle survey. The 3rd and 4th calls were open-ended, allowing the participant to select a diet or PA topic for discussion. For the weight loss maintenance RCT, participants were randomized with a 1:1 allocation ratio to receive either 36 phone contacts (weekly during months 1–6 and biweekly during months 7–12), or 18 phone contacts (biweekly during months 1–6, and monthly during months 7–12) as described in Section 3 of Additional file 1.

### High BP study intervention

The HHL high BP study [38] used a community based participatory research (CBPR) approach in local primary care practices to design and test a multilevel intervention with both a practice and patient component. The overall goal was to improve BP control rates and narrow disparities in systolic BP control between African Americans and Whites and those with lower and higher health literacy. At the practice level, providers and staff were engaged during intervention development and collaborated with the research team to design practice quality improvement strategies including: monthly design team calls, 10 regional dinner meetings to improve office-based BP management, on-site practice facilitation to guide practice change, embedding principles of health literacy in intervention processes and patient educational materials, and reviewing electronic health record hypertension performance data.

The patient component included a prospective cohort study enrolling 525 adults receiving care at these practices, 200 of whom also took part in the HHL lifestyle study. At the enrollment visit, participants received a BP monitor (Omron Model BP 785 or Omron BP 653 wrist monitor), were asked to record BP 3 times weekly, and received monthly 15-min phone coaching calls for a year. The coaches used scripted healthy lifestyle and hypertension management information, motivational interviewing techniques, and goal setting strategies to promote behavioral change. Curriculum topics covered: stress management, alcohol and tobacco use, healthy eating, PA, patient/provider interaction, medication adherence, and weight loss as previously described [38]. After each phone coaching session, a short session summary was faxed to the primary care provider. Of note, the phone coaching component of the high BP study began almost 6 months after the lifestyle study intervention such that only 13 participants in the lifestyle study received coaching phone calls before the 6 month follow-up visit.

### Measures

Outcome measures were assessed at baseline and 6, 12, and 24 month follow-up visits (except as noted). Previously validated questionnaires were administered addressing overall diet quality (DRA) [44, 62], fruit and vegetable intake [63], dietary fat quality [64], PA, [65, 66] and quality of life (SF-12 instrument, Quality Metric, Inc., Lincoln, RI). The DRA is a 26 item food frequncy questionnaire: each item contributes a score of 0, 1, or 2, which are summed for the total score, with range of 0–52. Similarly, the dietary fat quality questionnaire is comprized of 15 items: 3 about type of margarine are not scored with 12 scored as 0, 1, or 2, summed for a total score ranging from 0 to 24. Weight, as the average of two measures, was assessed in pounds to the nearest tenth by electronic scale (Seca 874, Seca, Hanover, MD) and height, measured at baseline only, was assessed with a portable stadiometer (Weigh and Measure, LLC, Olney, MD). After being seated for 5 min, 3 automated BP measurements were obtained (Omron HEM-907XL, Omron Healthcare, Lake Forest, IL) at 60 s intervals and averaged. Total and high-density lipoprotein cholesterol (HDL-C) were assessed at baseline, 12, and 24 months by enzymatic methods (LabCorp, Burlington, NC). Blood carotenoids (Molecular Epidemiology and Biomarker Research Laboratory, University of Minnesota, Minneapolis, MN) [62] were assessed at baseline, 6, and 12 months, as previously described [59]. At each follow-up visit, participants were queried about adverse events. Participants received $40 for enrollment, $25 for 6 and 12 month visits, and $30 for the 24 month visit.

### Sample size, randomization, and statistical analysis

The enrollment goal of 350 participants was based on having a sufficient sample (*N* = 100) for the embedded RCT of weight loss maintenance, assuming 60 % of study participants (*N* = 210) would choose to be in the weight loss intervention and 50 % of those (*N* = 105) would lose ≥ 8 lb [40]. An N of 100 in the RCT would provide 80 % power to detect a weight difference of 2 kg between study groups. In addition, the overall sample of 350 was felt sufficient to describe the primary objective of the intervention, to improve diet quality at 6 month follow-up, and the major secondary objectives of improved diet quality, PA, and weight loss at 12 and 24 month follow-up.

Study sample characteristics were summarized using descriptive statistics. Participants who became pregnant, had bariatric surgery, or were diagnosed with cancer (excluding non-melanoma skin cancer or localized breast or prostate cancer diagnosed by screening tests) were not included in the analysis of outcomes. Otherwise, pre-post changes in study outcomes were assessed among returnees, by intervention group, and by race using paired t-tests for continuous outcomes and McNemar’s tests for binary outcomes. The carotenoid index score was log-transformed for obtaining *p*-values from paired t-tests. To explore predictors of change in BP at follow-up, we used linear regression models that assessed change in BP associated with change in diet, PA, and weight, while controlling for selected baseline characteristics. As the RCT turned out to be substantially under powered (*N* = 27), weight change for RCT participants are described, but statistical tests comparing outcomes by group were not performed. SAS version 9.3 (SAS Institute, Cary, NC) was used for analyses, with *p* ≤ .05 considered statistically significant.

## Results

### Enrollment and baseline characteristics of participants

**Fig. 2:**
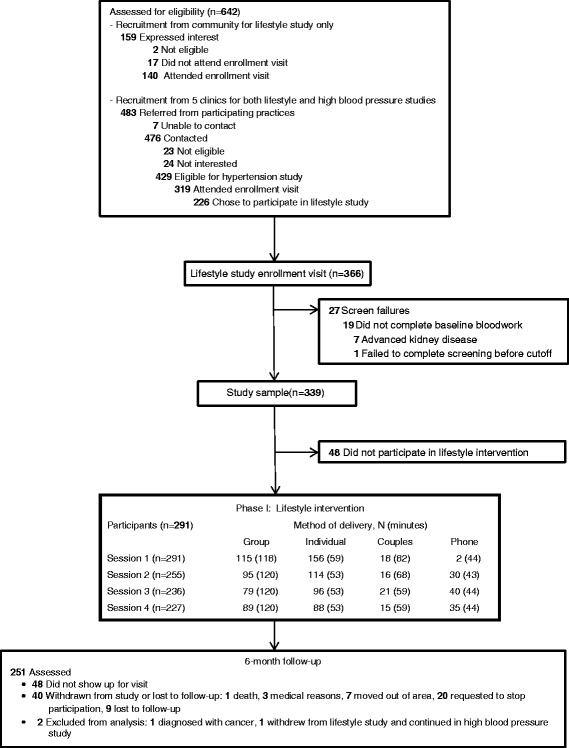
Study flow diagram, part 1

As depicted in Fig. 2, 339 participants completed baseline measures and comprised the study sample, with baseline characteristics presented in Table 2. The mean age was 56 years (range 18 to 92), 260 (77 %) were female, 219 (65 %) were African American, 124 (37 %) were employed full-time, 251 (74 %) had health insurance, 210 (62 %) had an annual household income of ≤ $40,000 per year, 291 (86 %) had hypertension, and the mean BMI was 36 kg/m^2^. As anticipated, the mean baseline blood pressure was substantially higher in participants taking part in both the lifestyle and high BP study as compared to those only taking part in the lifestyle study. On average, African American participants were younger, less educated, had lower household income, and with the exception of HDL-C had more CVD risk factors.

**Table 2:** Baseline characteristics: overall, by race, and by intervention group^a^

Characteristics	Overall	Race^b^		Participant selected intervention group^c^		
		African	White	Lifestyle	Weight loss, group	Weight loss, combination
		American				
	*n* = 339	*n* = 219	*n* = 117	*n* = 160	*n* = 57	*n* = 81
Demographics						
Age, mean (SE)	56 (0.6)	54 (0.8)	58 (1.0)	57 (1.0)	53 (1.3)	55 (1.1)
Female sex	260 (77)	181 (83)	76 (65)	110 (69)	52 (91)	72 (89)
Race						
African American				101 (63)	41 (72)	62 (79)
White				58 (37)	16 (28)	17 (21)
Education, y						
≤ 8 (middle school or less)	16 (5)	11 (5)	5 (4)	11 (7)	1 (2)	2 (3)
9–11 (some high school)	45 (13)	35 (16)	9 (8)	27 (17)	5 (9)	9 (11)
12 (high school graduate)	128 (38)	94 (43)	34 (29)	60 (37)	20 (35)	33 (41)
13–15 (some college)	79 (23)	45 (21)	33 (28)	35 (22)	15 (26)	18 (22)
16 (college graduate)	49 (14)	26 (12)	22 (19)	17 (11)	11 (19)	13 (16)
> 16 (graduate school)	22 (7)	8 (4)	14 (12)	10 (6)	5 (9)	6 (7)
Education: high school or less	189 (56)	140 (64)	48 (41)	98 (61)	26 (46)	44 (54)
Marital status						
Married or living with a partner	159 (47)	82 (37)	76 (65)	69 (43)	23 (40)	41 (51)
Other	180 (53)	137 (63)	41 (35)	91 (57)	34 (60)	40 (49)
Currently have health insurance	251 (74)	156 (71)	92 (79)	117 (73)	45 (79)	58 (72)
Current employment						
Working full time	124 (37)	82 (37)	40 (34)	46 (29)	29 (51)	38 (47)
Working part time	42 (12)	31 (14)	10 (8)	23 (14)	4 (7)	10 (12)
Do not work due to health reasons	69 (20)	43 (20)	26 (22)	37 (23)	9 (16)	16 (20)
Retired	53 (16)	26 (12)	27 (23)	29 (18)	7 (12)	8 (10)
Other	51 (15)	37 (17)	14 (12)	25 (16)	8 (14)	9 (11)
Annual household income						
< $10,000	62 (20)	50 (26)	11 (10)	40 (29)	6 (11)	9 (11)
$10,000 to < $20,000	64 (21)	45 (23)	19 (17)	29 (21)	11 (21)	18 (23)
$20,000 to < $40,000	84 (28)	60 (31)	23 (21)	32 (24)	14 (26)	27 (35)
$40,000 to < $60,000	33 (11)	15 (8)	18 (17)	11 (8)	8 (15)	9 (11)
$60,000 to < $80,000	27 (9)	13 (7)	14 (13)	11 (8)	11 (21)	5 (6)
≥ $80,000	34 (11)	10 (5)	24 (22)	13 (10)	3 (6)	10 (13)
CVD and risk factors for CVD						
Known coronary heart disease	49 (14)	30 (14)	19 (16)	29 (18)	5 (9)	5 (6)
Known CVD	62 (18)	37 (17)	25 (21)	34 (21)	7 (12)	8 (10)
Hypertension	291 (86)	195 (89)	95 (81)	143 (89)	47 (82)	66 (81)
Cholesterol category						
High (≥240 mg/dL)	187 (56)	110 (51)	76 (65)	88 (56)	31 (55)	47 (59)
Borderline (200–239 mg/dL)	46 (14)	33 (15)	12 (10)	16 (10)	11 (19)	13 (16)
Desirable (<200 mg/dL)	102 (30)	72 (33)	29 (25)	54 (34)	15 (26)	20 (25)
Diabetes	124 (37)	89 (41)	34 (29)	62 (39)	21 (37)	27 (33)
Current cigarette smoker	54 (16)	37 (17)	17 (14)	36 (22)	4 (7)	5 (6)
Packs of cigarettes smoked per day, mean (SE) for current smokers	0.7 (0.1)	0.6 (0.1)	0.9 (0.1)	0.7 (0.1)	0.6 (0.2)	0.6 (0.3)
Taking BP lowering medication	260 (77)	176 (80)	83 (71)	129 (81)	43 (75)	60 (74)
Lifestyle mean (SE)						
DRA total score	27.8 (0.3)	28.0 (0.5)	27.6 (0.4)	27.8 (0.5)	27.2 (0.7)	27.6 (0.6)
Fat quality screener score	15.5 (0.2)	15.4 (0.2)	15.07 (0.3)	15.4 (0.2)	15.3 (0.3)	15.4 (0.3)
Fruit and vegetable, servings per day	3.4 (0.1)	3.4 (0.1)	3.5 (0.2)	3.5 (0.2)	3.0 (0.3)	3.5 (0.2)
Carotenoid index^e^, mean (SE)	40.7 (1.3)	41.9 (1.6)	38.2 (2.5)	41.1 (2.0)	42.1 (3.5)	39.1 (2.4)
Walking time, min/wk^f^	91 (11.3)	100 (16.2)	73 (11.8)	110 (18.8)	97 (26.5)	55 (13.3)
Activity time, min/wk^g^	149 (14.0)	150 (18.2)	143 (21.5)	160 (22.7)	148 (29.1)	135 (25.7)
Physiologic mean (SE)						
Weight, kg	98.1 (1.4)	100 (1.7)	94 (2.3)	93.8 (2.0)	101.3 (3.3)	106.4 (2.6)
BMI, kg/m^2^	36 (0.5)	37 (0.6)	34 (0.8)	34 (0.7)	38 (1.1)	40 (1.0)
Systolic BP, mm Hg^h^	135 (1.2)	136 (1.6)	132 (1.8)	138 (1.8)	130 (2.6)	133 (2.3)
LS study only (*n* = 139)	125 (1.3)	126 (1.6)	122 (2.2)	124 (2.5)	126 (2.7)	126 (2.0)
LS and HBP study (*n* = 200)	142 (1.7)	143 (2.2)	139 (2.4)	142 (2.2)	137 (5.2)	142 (4.0)
Diastolic BP, mm Hg	82 (0.7)	83 (0.9)	80 (1.0)	83 (1.0)	82 (1.4)	81 (1.3)
LS study only (*n* = 139)	79 (0.8)	80 (1.0)	76 (1.4)	77 (1.5)	80 (1.5)	79 (1.5)
LS and HBP study (*n* = 200)	84 (0.9)	86 (1.3)	83 (1.3)	85 (1.2)	87 (2.7)	84 (2.2)
HbA_1c_, % of total Hb	6.6 (0.1)	6.7 (0.1)	6.2 (0.1)	6.5 (0.1)	6.9 (0.3)	6.5 (0.2)
Total cholesterol, mg/dL	193 (2.3)	191 (2.8)	196 (4.0)	191 (3.1)	198 (6.0)	189 (4.7)
HDL cholesterol, mg/dL	54 (0.8)	57 (1.0)	50 (1.4)	55 (1.2)	53 (1.8)	53 (1.5)

*Abbreviations: BMI* body mass index, *BP* blood pressure, *CVD* cardiovascular disease, *DRA* Dietary Risk Assessment, *HDL-C* high-density lipoprotein, *HbA*_*1c*_ hemoglobin A_1c_, *HBP* high blood pressure, *LS* lifestyle, *SE* standard error

*Note*: SI unit conversion factors: To convert all types of cholesterol to millimoles per liter, multiply by 0.0259

^a^ Unless otherwise noted, data are reported as number (percentage) of participants

^b^ 3 categorized as other race

^c^ Participants selected intervention group at 6 month follow-up visit; if they did not attend this visit they were assigned to lifestyle group

^d^ Framingham risk scores calculated as percent chance of developing angina, having a myocardial infarction, or dying of coronary heart disease over a 10 year time frame

^e^ Carotenoid index, calculated as the sum of α-carotene, β-carotene,β-cryptoxanthin, and zeaxanthin. Data presented are for nonsmokers (*n* = 261). A higher index indicates greater fruit and vegetable consumption. Statistical tests performed on log-transformed data

^f^ Includes walking for transportation and exercise

^g^ Includes walking and other moderate and vigorous activity

^h^ Systolic and diastolic blood pressure results are also presented for participants in LS study only and participants in both LS and HBP studies

### Phase I outcomes

As outlined in Fig. 2, 291 (86 %) participants attended the first intervention visit, with 115 (40 %) choosing group format, and the rest individual counseling. Overall, 237 (70 %) completed all counseling sessions. As shown in Table 3, from baseline to 6 month follow-up, there were improvements in self-reported measures of diet quality (higher score indicates improved diet quality): DRA score increased 4.4 units (95 % CI 3.7 to 5.0), fat quality score increased 1.4 units (1.0 to 1.7), and servings of fruit and vegetables per day increased by 0.3 (0.1 to 0.5). In addition, there were increases in walking of 64 min/week (19 to 110) and total activity time (walking plus other types of moderate to vigorous PA) of 97 min/week (36 to 158). For physiologic outcomes, change in systolic BP was −6.4 mmHg (−8.7 to −4.1), diastolic BP −3.7 mmHg (−4.9 to −2.5), and weight −0.7 kg (−1.2 to −0.3). Of note, there were significant reductions in systolic, −4.7 mmHg (−6.9 to −2.6) and diastolic, −2.8 mmHg (−4.2 to −1.3) BP in the lifestyle only group. Among those who took part in both the lifestyle and high BP studies the systolic, −7.9 mmHg (−11.8 to 4.0), and diastolic, −4.6 mmHg (−6.5 to −2.7), BP reductions were larger. In addition, desirable outcomes were similar or larger for African Americans compared to whites.

**Table 3:** Change from baseline to 6 months^a^

Outcome	Phase 1	
	Baseline to 6 months	
	(6 months minus baseline)	
	n	Mean, 95 % CI
Dietary		
DRA total score		
All	235	**4.3 (3.7 to 5.0)** ^***^
Subgroup by race^b^		
--African American	155	**4.6 (3.7 to 5.5)** ^***^
--White	77	**3.9 (2.9 to 5.0)** ^***^
Fat quality screener score		
All	229	**1.4 (1.0 to 1.7)** ^***^
--African American	150	**1.4 (0.9 to 1.8)** ^***^
--White	76	**1.3 (0.7 to 2.0)** ^***^
Fruit and vegetable servings per day		
All	249	**0.3 (0.1 to 0.5)** ^**^
--African American	168	0.2 (0.0 to 0.5)
--White	78	**0.3 (0.0 to 0.7)** ^*^
Carotenoid index^c^		
All	169	−0.9 (−3.3 to 1.5)
--African American	118	−0.2 (−2.8 to 2.5)
--White	49	−3.5 (−8.7 to 1.8)
Physical Activity		
Walking time, min/wk^d^		
All	249	**64 (19 to 109)** ^**^
--African American	168	**65 (5 to 125)** ^*^
--White	78	**66 (4 to 128)** ^*^
Activity time, min/wk^e^		
All	249	**97 (36 to 158)** ^**^
--African American	168	**106 (28 to 184)** ^**^
--White	78	88(−13 to 188)
Blood Pressure Medication		
Taking BP lowering medication, %		
All	249	2.0 % (−0.3 to 4.4)
--African American	167	1.2 % (−1.7 to 4.1)
--White	78	3.8 % (−0.4 to 8.1)
Physiologic		
Systolic BP, mm Hg^f^		
*LS study only*	119	**−4.7 (−6.9 to −2.6)** ^***^
*LS and HBP study*	130	**−7.9 (−11.8 to 4.0)** ^***^
All	249	**−6.4 (−8.7 to −4.1)** ^***^
--African American	168	**−6.8 (−9.7 to −3.8)** ^***^
--White	78	**−5.6 (−9.4 to −1.8)** ^**^
Diastolic BP, mm Hg		
*LS study only*	119	**−2.8 (−4.2 to −1.3)** ^***^
*LS and HBP study*	130	**−4.6 (−6.5 to −2.7)** ^***^
All	249	**−3.7 (−4.9 to −2.5)** ^***^
--African American	168	**−3.5 (−5.1 to −2.0)** ^***^
--White	78	**−4 (−6.1 to −2.3)** ^***^
Weight, kg		
All	248	**−0.7 (−1.2 to −0.3)** ^**^
-African American	167	**−0.8 (−1.4 to −0.3)** ^**^
--White	78	−0.4 (−1.3 to 0.5)
Weight, ≥ 5 % weight loss, %		
All	248	9 % (6 to 13)
--African American	167	12 % (7 to 17)
--White	78	4 % (0 to 8)

*Abbreviations: BP* blood pressure, *CI* confidence interval, *DRA* dietary risk assessment, *HBP* high blood pressure, *LS* lifestyle

*Note:* Boldface indicates statistical significance ^*^ *p* ≤ 0.05; ^**^ *p* ≤ 0.01; ^***^ *p* ≤ 0.001

^a^ Data are means except where noted

^b^ 3 categorized as other race

^c^ Carotenoid index, calculated as the sum of α-carotene, β-carotene,β-cryptoxanthin, and zeaxanthin. Data presented are for nonsmokers and available only through 12 month follow-up visit. A higher index indicates greater fruit and vegetable consumption. Statistical tests performed on log-transformed data

^d^ Includes walking for transportation and exercise

^e^ Includes walking and other moderate and vigorous activity

^f^ Systolic and diastolic blood pressure results are also presented for participants in LS study only and participants in both LS and HBP studies

Of 242 participants completing the Phase I acceptability questionnaire, 170 (70 %) strongly agreed and 66 (27 %) agreed with the following statement: “I would recommend the program to others.” Similarly, 138 (57 %) strongly agreed and 99 (41 %) agreed that “the information provided was easy to understand.” In terms of diet counseling content, the information on nuts, spreads, dressings and oils was considered most helpful with 185 (76 %) indicating this session was very helpful and 43 (18 %) somewhat helpful.

### Phase II outcomes

**Fig. 3:**
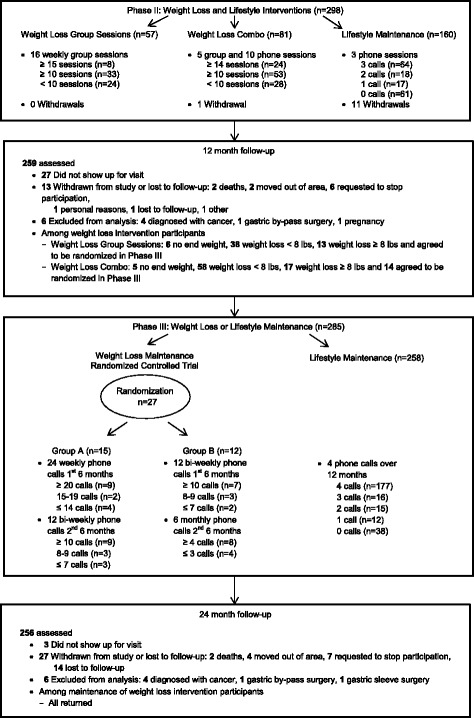
Study flow diagram, part 2

Figure 3 shows 298 (88 %) participants continued into Phase II: of these, 57 chose the group-based weight loss and 81 the combination format, with 160 receiving the lifestyle maintenance intervention (of these, 111 attended the 6 month measurement visit and 93 (84 %) had BMI ≥ 25 kg/m^2^ but did not choose to be in the weight loss program). Change in outcomes from 6 to 12 and baseline to 12 months are shown in Tables 4 and 5. Self-reported dietary changes were generally maintained through 12 months, but more so for the weight loss groups, especially for fruit and vegetable intake. From 6 to 12 months, the carotenoid index increased significantly for whites and from baseline to 12 months, there was a statistically significant increase in the carotenoid index score for all participants. The increase in PA reported from baseline to 6 months was generally maintained at 12 months, though less so for the group-based weight loss participants. Change in BP from baseline to 12 months among participants in the lifestyle only study was: systolic, −1.4 mmHg (−4.5 to 1.6); diastolic −2.0 mmHg (−3.7 to −0.4). For those in both studies, it was: systolic, −9.3 mmHg (−13.6 to −5.1); diastolic −7.0 mmHg (−9.0 to −4.9). At 12 months, among all participants there was a significant change in HDL-C of −2 mg/dL (−2.8 to −0.4) and a non-significant change in total cholesterol of −3 mg/dL (−7.0 to 0.7).

**Table 4:** Change in dietary and physical activity outcomes at 12 months^a^

Outcome	Phase 2			
	6 months to 12 months		Baseline to 12 months	
	(12 months minus 6 months)		(12 months minus baseline)	
	n	Mean, 95 % CI	n	Mean, 95 % CI
Dietary				
DRA total score				
All	219	**−0.7 (−1.3 to −0.1)** ^*^	227	**3.3 (2.5 to 4.0)** ^***^
Subgroup by race^b^				
--African American	151	−0.2 (−1.0 to 0.6)	156	**4.0 (3.1 to 4.9)** ^***^
--White	66	**−1.9 (−2.7 to −1.1)** ^***^	69	**1.7 (0.4 to 3.0)** ^**^
Subgroup by intervention^c^				
--Lifestyle	93	0.0 (−1.0 to 0.9)	102	**1.7 (0.7 to 2.8)** ^***^
--Wt loss, group	51	−0.3 (−1.5 to 0.9)	51	**5.1 (3.8 to 6.4)** ^***^
--Wt loss, combination	75	**−1.7 (−2.8 to −0.7)** ^***^	74	**4.1 (2.7 to 5.6)** ^***^
Fat quality screener score				
All	218	−0.1 (−0.5 to 0.2)	225	**1.0 (06 to 1.3)** ^***^
--African American	150	0.1 (−0.3 to 0.5)	154	**1.2 (0.8 to 1.7)** ^***^
--White	66	**−0.6 (−1.2 to −0.1)** ^*^	69	0.4 (−0.2 to 1.0)
--Lifestyle	93	0.2 (−0.4 to 0.8)	102	**0.7 (0.1 to 1.2)** ^*^
--Wt loss, group	51	−0.1 (−0.8 to 0.5)	48	**1.3 (0.6 to 2.0)** ^***^
--Wt loss, combination	74	**−0.6 (−1.1 to −0.1)** ^*^	75	**1.2 (0.6 to 1.8)** ^***^
Fruit and vegetable servings per day				
All	221	**0.4 (0.2 to 0.5)** ^***^	253	**0.5 (0.3 to 0.8)** ^***^
--African American	153	**0.5 (0.2 to 0.7)** ^***^	178	**0.5 (0.2 to 0.9)** ^**^
--White	66	0.0 (−0.3 to 0.3)	73	**0.5 (0.1 to 0.9)** ^**^
--Lifestyle	95	0.1 (−0.2 to 0.4)	127	0.1 (−0.2 to 0.5)
--Wt loss, group	51	**0.5 (0.1 to 0.9)** ^*^	51	**1.0 (0.5 to 1.5)** ^***^
--Wt loss, combination	75	**0.6 (0.2 to 0.9)** ^***^	75	**0.9 (0.4 to 1.3)** ^***^
Carotenoid index^d^				
All	105	3.7 (0.4 to 6.9)	117	**3.1 (−0.4 to 6.7)** ^*^
--African American	77	3.1 (−0.8 to 7.1)	88	3.0 (−1.4 to 7.5)
--White	27	**5.6 (−0.4 to 11.7)** ^*^	28	3.4 (−2.0 to 8.9)
--Lifestyle	33	5.6 (−0.2 to 11.9)	40	3.1 (−3.2 to 9.5)
--Wt loss, group	29	1.9 (−3.5 to 7.3)	31	3.7 (−3.0 to 10.4)
--Wt loss, combination	43	3.2 (−2.0 to 8.4)	46	2.7 (−2.9 to 8.3)
Physical Activity				
Walking time, min/wk^e^				
All	221	7 (−49 to 63)	253	**71 (28 to 113)** ^***^
--African American	153	16 ((−59 to 91)	178	**68 (13 to 123)** ^*^
--White	66	−14 (−88 to 61)	73	**82 (23 to 142)** ^**^
--Lifestyle	95	29 (−56 to 113)	127	60 (−10 to 130)
--Wt loss, group	51	−21 (−164 to 123)	51	66 (−27 to 158)
--Wt loss, combination	75	−2 (−81 to 77)	75	**93 (45 to 141)** ^***^
Activity time, min/wk^f^				
All	221	−26 (−96 to 44)	253	**83 (30 to 136)** ^**^
--African American	153	−19 (−115 to 77)	178	**75 (14 to 136)** ^*^
--White	66	−42 (−115 to 32)	73	**110 (2 to 218)** ^*^
--Lifestyle	95	3 (−84 to 90)	127	67 (−24 to 158)
--Wt loss, group	51	−90 (−310 to 131)	51	**108 (9 to 208)** ^*^
--Wt loss, combination	75	−19 (−105 to 66)	75	**93 (35 to 151)** ^**^

*Abbreviations: BP* blood pressure, *CI* confidence interval, *DRA* dietary risk assessment

*Note:* Boldface indicates statistical significance ^*^ *p* ≤ 0.05; ^**^ *p* ≤ 0.01; ^***^ *p* ≤ 0.001

^a^ Data are means except where noted

^b^ 3 categorized as other race

^c^ Participants selected intervention group at 6 month follow-up visit; if they did not attend this visit they were assigned to lifestyle group

^d^ Carotenoid index, calculated as the sum of α-carotene, β-carotene,β-cryptoxanthin, and zeaxanthin. Data presented are for nonsmokers and available only through 12 month follow-up visit. A higher index indicates greater fruit and vegetable consumption. Statistical tests performed on log-transformed data

^e^ Includes walking for transportation and exercise

^f^ Includes walking and other moderate and vigorous activity

**Table 5:** Change in blood pressure medication and physiologic outcomes at 12 months^a^

Outcome	Phase 2			
	6 months to 12 months		Baseline to 12 months	
	(12 months minus 6 months)		(12 months minus baseline)	
	n	Mean, 95 % CI	n	Mean, 95 % CI
Blood Pressure Medication				
Taking BP lowering medication, %				
All	221	−0.5 % (−2.8 to 1.9)	253	0.0 % (−3.3 to 3.3)
--African American	153	0.0 % (−3.1 to 3.1)	178	−0.6 % (−4.5 to 3.4)
--White	66	−1.5 % (−4.5 to 1.4)	73	1.4 % (−4.6 to 7.4)
--Lifestyle	95	−1.0 % (−3.1 to 1.0)	127	−0.8 % (−5.4 to 3.8)
--Wt loss, group	51	−2.0 % (−8.6 to 4.7)	51	−2.0 % (−10.5 to 6.6)
--Wt loss, combination	75	1.3 % (−3.2 to 5.8)	75	2.7 % (−2.5 to 7.9)
Physiologic				
Systolic BP, mm Hg				
LS study only	99	**3.2 (0.3 to 6.2)** ^*^	101	−1.4 (−4.5 to 1.6)
LS and HBP study	121	−2.5 (−6.5 to 1.4)	150	**−9.3 (−13.6 to −5.1)** ^***^
All	220	0.0 (−2.5 to 2.6)	251	**−6.1 (−9.0 to −3.3)** ^***^
Subgroup by race^b^				
--African American	152	−0.9 (−4.0 to 2.1)	176	**−6.9 (−10.5 to −3.3)** ^***^
--White	66	2.5 (−2.5 to 7.4)	73	−4.2 (−8.8 to 0.3)
Subgroup by intervention^c^				
--Lifestyle	95	−2.2 (−6.2 to 1.8)	126	**−8.3 (−12.6 to −3.9)** ^***^
--Wt loss, group	50	3.3 (−1.0 to 7.6)	50	−3.6 (−7.8 to 0.5)
--Wt loss, combination	75	0.7 (−3.8 to 5.3)	75	−4.3 (−9.7 to 1.1)
Diastolic BP, mm Hg				
LS study only	99	0.4 (−1.4 to 2.2)	101	**−2.0 (−3.7 to −0.4)** ^*^
LS and HBP study	121	**−3.1 (−5.3 to −1.0)** ^**^	150	**−7.0 (−9.0 to −4.9)** ^***^
All	220	**−1.5 (−2.9 to −0.1)** ^*^	251	**−5.0 (−6.4 to −3.6)** ^***^
--African American	152	**−2.3 (−4.0 to −0.5)** ^**^	176	**−5.2 (−7.0 to −3.4)** ^***^
--White	66	0.7 (−1.8 to 3.2)	73	**−4.2 (−6.4 to −1.9)** ^***^
--Lifestyle	95	−2.2 (−4.4 to 0.1)	126	**−5.4 (−7.5 to −3.2)** ^***^
--Wt loss, group	50	1.2 (−1.0 to 3.4)	50	**−4.2 (−6.9 to −1.6)** ^**^
--Wt loss, combination	75	−2.5 (−5.1 to 0.1)	75	**−4.5 (−7.2 to −2.3)** ^***^
Weight, kg				
All	219	**−1.1 (−1.7 to −0.5)** ^***^	250	**−1.7 (−2.5 to 1.0)** ^***^
--African American	151	**−0.9 (−1.6 to −0.3)** ^**^	175	**−1.7 (−2.6 to −0.8)** ^***^
--White	66	**−1.4 (−2.3 to −0.3)** ^*^	73	**−1.6 (−3.1 to −0.2)** ^*^
--Lifestyle	94	−0.3 (−1.3 to 0.6)	125	−0.9 (−2.1 to 0.2)
--Wt loss, group	50	**−1.3 (−2.4 to −0.2)** ^*^	50	**−3.1 (−4.9 to −1.3)** ^***^
--Wt loss, combination	75	**−1.9 (−2.8 to −1.0)** ^***^	75	**−2.1 (−3.2 to −1.0)** ^***^
Weight, ≥ 5 % weight loss, %				
All	219	18 % (13 to 23)	250	23 % (18 to 28)
--African American	151	18 % (12 to 24)	175	23 % (17 to 30)
--White	66	18 % (9 to 28)	73	22 % (12 to 31)
--Lifestyle	94	14 % (7 to 21)	125	19 % (12 to 26)
--Wt loss, group	50	22 % (10 to 34	50	34 % (21 to 47)
--Wt loss, combination	75	21 % (12 to 31)	75	23 % (13 to 32)
Total cholesterol, mg/dL^d^				
All			221	−3 (−7.0 to 0.7)
--African American			155	−4 (−8.5 to 0.4)
--White			64	0 (−7.8 to 7.7)
--Lifestyle			106	−3 (−9.1 to 2.7)
--Wt loss, group			44	−6 (−13.5 to 1.4)
--Wt loss, combination			71	−1 (−8.0 to 5.2)
HDL cholesterol, mg/dL				
All			220	**−2 (−2.8 to −0.4)** ^**^
--African American			154	**−2 (−3.0 to 0)** ^*^
--White			64	−2 (−3.8 to 0.3)
--Lifestyle			106	−2 (−3.8 to −0.1)
--Wt loss, group			44	−1 (−3.1 to 1.5)
--Wt loss, combination			70	−1 (−3.5 to 0.5)

*Abbreviations: BP* blood pressure, *CI* confidence interval, *HBP* high blood pressure, *LS* lifestyle

*Note:* Boldface indicates statistical significance ^*^ *p* ≤ 0.05; ^**^ *p* ≤ 0.01; ^***^ *p* ≤ 0.001.

SI unit conversion factors: To convert all types of cholesterol to millimoles per liter, multiply by 0.0259

^a^ Data are means except where noted

^b^ 3 categorized as other race

^c^ Participants selected intervention group at 6 month follow-up visit; if they did not attend this visit they were assigned to lifestyle group

^d^ To convert from mg/dL to mmol/L divide by 68.37

From baseline to 12 months, weight change was −0.9 kg (−2.1 to 0.2) for the lifestyle maintenance intervention, with 24 (19 %) losing ≥ 5 % body weight; −3.1 kg (−4.9 to −1.3) for the group-based weight loss intervention, with 17 (34 %) losing ≥ 5 % body weight; and −2.1 kg (−3.2 to −1.0) for the combination weight loss intervention, with 17 (23 %) ≥ 5 % body weight. During Phase II, outcomes were similar for African Americans and Whites. At the completion of Phase II, 46 participants in the group-based and 70 in the combination weight loss intervention completed an acceptability survey. Among group participants, 37 (80 %) reported they were very satisfied and 6 (13 %) reported they were satisfied with the intervention. For combination participants, 51 (71 %) reported they were very satisfied and 12 (17 %) reported they were satisfied with the intervention.

### Phase III outcomes

Of the 30 participants who lost ≥ 8 lb at 12 month follow-up, 27 (90 %) agreed to take part in the maintenance of weight loss RCT, while 258 received the lifestyle maintenance intervention (Fig. 3). Among those in the RCT, compared to baseline, weight change was −7.0 kg at 12 and −5.9 kg at 24 months for the 15 participants in the intensive intervention and −6.9 kg and −2.4 kg, respectively for the 12 participants in the less intensive intervention. Those losing ≥ 5 % body weight at 24 months included 7 (47 %) receiving the more intensive and 3 (25 %) receiving the less intensive intervention.

Change in outcomes from 12 to 24 and baseline to 24 months for all returnees are shown in Tables 6 and 7. In general, during the 2nd year of the intervention, there was minor attenuation in self-reported dietary behaviors and more pronounced attenuation of PA. Change in BP from baseline to 24 months among participants in the lifestyle only study was: systolic, −4.2 (−7.3 to 1.2); diastolic −5.2 (−7.1 to −3.3). For those in both studies, it was: systolic, −9.4 (−13.4 to −5.4); diastolic −7.8 (−10.1 to −5.5). Compared to baseline, weight change was −1.7 kg (−2.9 to −0.5) for the lifestyle only intervention with 29 (23 %) losing ≥ 5 % body weight, −2.1 kg (−4.3 to 0.0) for the group weight loss intervention with 13 (25 %) losing ≥ 5 % body weight, and −1.1 kg (−2.7 to 0.4) for the combination weight loss intervention with 15 (21 %) ≥ 5 % body weight. During Phase III, outcomes were similar for African Americans and Whites. Change in blood lipids at 24 month was minimal.

**Table 6:** Change in dietary and physical activity outcomes at 24 months^a^

Outcome	Phase 3			
	12 months to 24 months		Baseline to 24 months	
	(24 months minus 12 months)		(24 months minus baseline)	
	n	Mean, 95 % CI	n	Mean, 95 % CI
Dietary				
DRA total score				
All	236	−0.3 (−0.8 to 0.2)	226	**2.9 (2.3 to 3.6)** ^***^
Subgroup by race^b^				
--African American	168	**−0.7 (−1.3 to 0.0)** ^*^	158	**3.0 (2.2 to 3.8)** ^***^
--White	66	0.6 (−0.2 to 1.4)	66	**2.8 (1.6 to 4.0)** ^***^
Subgroup by intervention^c^				
--Lifestyle	116	−0.1 (−0.8 to 0.6)	105	**1.8 (0.9 to 2.7)** ^***^
--Wt loss, group	48	**−1.1 (−2.1 to −0.2)** ^*^	50	**4.0 (2.7 to 5.4)** ^***^
--Wt loss, combination	72	0.0 (−0.9 to 1.0)	71	**3.9 (2.6 to 5.2)** ^***^
Fat quality screener score				
All	236	**−0.4 (−0.7 to 0)** ^*^	224	**0.7 (0.3 to 1.0)** ^***^
--African American	168	**−0.6 (−1.0 to −0.1**)^**^	156	**0.7 (0.2 to 1.1)** ^**^
--White	66	0.1 (−0.5 to 0.6)	66	0.6 (−0.2 to 1.5)
--Lifestyle	116	**−0.7 (−1.3 to −0.2)** ^**^	105	0.1 (−0.5 to 0.7)
--Wt loss, group	48	−0.2 (−0.9 to 0.5)	47	**1.2 (0.4 to 2.0)** ^**^
--Wt loss, combination	72	0.1 (−0.5 to 0.6)	72	**1.1 (0.5 to 1.7)** ^***^
Fruit and vegetable servings per day				
All	237	−0.2 (−0.4 to 0.1)	250	**0.4 (0.2 to 0.6)** ^***^
--African American	169	−0.2 (−0.5 to 0.1)	177	**0.3 (0.1 to 0.6)** ^*^
--White	66	0.0 (−0.4 to 0.3)	71	**0.5 (0.1 to 0.8)** ^**^
--Lifestyle	117	0.1 (−0.2 to 0.4)	127	0.2 (−0.1 to 0.5)
--Wt loss, group	48	−0.1 (−0.6 to 0.3)	51	**0.9 (0.4 to 1.4)** ^***^
--Wt loss, combination	72	**−0.5 (−0.9 to −0.1)** ^**^	72	0.3 (−0.1 to 0.8)
Physical Activity				
Walking time, min/wk^d^				
All	237	**−52 (−89 to −15)** ^**^	250	22 (−13 to 56)
--African American	169	**−49 (−95 to −3)** ^*^	177	21 (−25 to 67)
--White	66	**−63 (−123 to −2)** ^*^	71	27 (−12 to 65)
--Lifestyle	117	−55 (−111 to 2)	127	8 (−50 to 65)
--Wt loss, group	48	**−81 (−163 to 0)** ^*^	51	−4 (−69 to 60)
--Wt loss, combination	72	−29 (−84 to 27)	72	**65 (22 to 108)** ^**^
Activity time, min/wk^e^				
All	237	−40 (−91 to 11)	250	48 (−7 to 103)
--African American	169	−25 (−89 to 40)	177	54 (−17 to 124)
--White	66	**−82 (−164 to −0.5)** ^*^	71	39 (−46 to 124)
--Lifestyle	117	−17 (−106 to 73)	127	51 (−46 to 149)
--Wt loss, group	48	**−116 (−208 to −24)** ^**^	51	12 (−70 to 95)
--Wt loss, combination	72	−27 (−84 to 29)	72	**68 (7 to 129)** ^*^

*Abbreviations: BP* blood pressure, *CI* confidence interval, *DRA* dietary risk assessment

*Note:* Boldface indicates statistical significance ^*^ *p* ≤ 0.05; ^**^ *p* ≤ 0.01; ^***^ *p* ≤ 0.001

^a^ Data are means except where noted

^b^ 3 categorized as other race

^c^ Participants selected intervention group at 6 month follow-up visit; if they did not attend this visit they were assigned to lifestyle group

^d^ Includes walking for transportation and exercise

^e^ Includes walking and other moderate and vigorous activity

**Table 7:** Change in blood pressure medication and physiologic outcomes at 24 months^a^

Outcome	Phase 3			
	12 months to 24 months		Baseline to 24 months	
	(24 months minus 12 months)		(24 months minus baseline)	
	n	Mean, 95 % CI	n	Mean, 95 % CI
Blood Pressure Medication				
Taking BP lowering medication, %				
All	237	0.4 % (−2.3 to 3.2)	250	0.8 % (−3.0 to 4.6)
--African American	169	1.2 %(−1.6 to 4.0)	177	1.1 % (−3.3 to 5.6)
--White	66	0.0 % (−5.9 to 5.9)	71	1.4 % (−5.9 to 8.7)
--Lifestyle	117	2.6 % (−1.1 to 6.3)	127	1.6 % (−4.2 to 7.3)
--Wt loss, group	48	2.1 % (−5.0 to 9.1)	51	2.0 % (−6.6 to 10.5)
--Wt loss, combination	72	−4.2 % (−8.8 to 0.4)	72	−1.4 % (−7.5 to 4.7)
Physiologic				
Systolic BP, mm Hg				
LS study only	95	−2.8 (−6.4 to 0.7)	104	**−4.2 (−7.3 to 1.2)** ^**^
LS and HBP study	140	−0.2 (−3.8 to 3.5)	146	**−9.4 (−13.4 to −5.4)** ^***^
All	235	−1.3 (−3.9 to 1.3)	250	**−7.2 (−9.9 to −4.6)** ^***^
Subgroup by race^b^				
--African American	167	−2.0 (−5.3 to 1.2)	177	**−8.4 (−11.8 to −5.1)** ^***^
--White	66	0.9 (−3.3 to 5.2)	71	−4.1 (−8.5 to 0.4)
Subgroup by intervention^c^				
--Lifestyle	116	−1.2 (−4.9 to 2.5)	127	**−8.8 (−12.9 to −4.8)** ^***^
--Wt loss, group	47	0.4 (−3.9 to 4.8)	51	−3.9 (−8.4 to 0.6)
--Wt loss, combination	72	−2.4 (−7.8 to 2.9)	72	**−6.8 (−11.8 to −1.9)** ^**^
Diastolic BP, mm Hg				
*LS study only*	95	**−3.4 (−5.4 to −1.5)** ^***^	104	**−5.2 (−7.1 to −3.3)** ^***^
*LS and HBP study*	140	−0.9 (−2.9 to 1.0)	146	**−7.8 (−10.1 to −5.5)** ^***^
All	235	−1.9 (−3.4 to −0.5)	250	**−6.7 (−8.3 to −5.1)** ^***^
--African American	167	**−2.4 (−4.2 to 0.5)** ^**^	177	**−7.2 (−9.2 to −5.1)** ^***^
--White	66	−1.0 (−3.0 to 1.0)	71	**−5.4 (−7.6 to −3.3)** ^***^
--Lifestyle	116	−1.9 (−4.0 to 0.2)	127	**−6.8 (−9.1 to −4.4)** ^***^
--Wt loss, group	47	**−3.1 (−5.8 to −0.4)** ^*^	51	**−7.7 (−10.7 to −4.6)** ^***^
--Wt loss, combination	72	−1.3 (−3.9 to 1.3)	72	**−6.0 (−8.8 to −3.2)** ^***^
Weight, kg				
All	232	0.1 (−0.6 to 0.8)	247	**−1.6 (−2.5 to −0.7)** ^***^
--African American	164	−0.1 (−1.0 to .08)	174	**−1.8 (−2.8 to −0.8)** ^***^
--White	66	0.6 (−0.6 to 1.7)	71	−1.0 (−2.9 to 0.8)
--Lifestyle	113	−0.9 (−2.0 to 0.1)	124	**−1.7 (−2.9 to −0.5)** ^**^
--Wt loss, group	47	1.2 (−0.4 to 2.8)	51	**−2.1 (−4.3 to 0.0)** ^*^
--Wt loss, combination	72	1.0 (−0.2 to 2.2)	72	−1.1 (−2.7 to 0.4)
Weight, ≥ 5 % weight loss, %				
All	232	12 % (8 to 16)	247	23 % (18 to 28)
--African American	164	12 % (7 to 17)	174	24 % (18 to 31)
--White	66	12 % (4 to 20)	71	18 % (9 to 27)
--Lifestyle	113	16 % (9 to 23)	124	23 % (16 to 31)
--Wt loss, group	47	11 % (2 to 20)	51	25 % (13 to 38)
--Wt loss, combination	72	7 % (1 to 13)	72	21 % (11 to 30)
Total cholesterol, mg/dL^d^				
All	196	1 (−3.3 to 5.0)	211	−3 (−7.8 to 1.7)
--African American	139	−1 (−6.3 to 4.7)	148	−4 (−10.3 to 1.4)
--White	55	4 (−0.9 to 9.8)	61	1 (−7.3 to 9.2)
--Lifestyle	91	2 (−4.0 to 8.8)	99	−2 (−8.1 to 4.3)
--Wt loss, group	41	−3 (−13.7 to 7.2)	46	−9 (−20.9 to 3.7)
--Wt loss, combination	64	1 (−4.7 to 7.1)	66	−1 (−9.3 to 7.2)
HDL cholesterol, mg/dL				
All	196	0 (−0.8 to 1.3)	211	−1 (−2.3 to 0.3)
--African American	139	0 (−1.2 to 1.6)	148	−1 (−2.6 to 0.6)
--White	55	0 (−1.4 to 2.2)	61	−1 (−3.1 to 1.2)
--Lifestyle	91	0 (−1.4 to 1.7)	99	**−2 (−4.0 to −0.1)** ^*^
--Wt loss, group	41	0 (−2.0 to 2.3)	46	1 (−1.6 to 3.6)
--Wt loss, combination	64	0 (−1.6 to 2.4)	66	−1 (−2.9 to 1.4)

*Abbreviations: BP* blood pressure, *CI* confidence interval, *HBP* high blood pressure, *LS* lifestyle

*Note:* Boldface indicates statistical significance ^*^ *p* ≤ 0.05; ^**^ *p* ≤ 0.01; ^***^ *p* ≤ 0.001

SI unit conversion factors: To convert all types of cholesterol to millimoles per liter, multiply by 0.0259

^a^ Data are means except where noted

^b^ 3 categorized as other race

^c^ Participants selected intervention group at 6 month follow-up visit; if they did not attend this visit they were assigned to lifestyle group

^d^ To convert from mg/dL to mmol/L divide by 68.37

### Predictors of BP change, quality of life, and adverse events

At all follow-up time points, we examined the association between change in BP as a function of change in diet, PA, or weight, controlling for the baseline value of BP and baseline assessment of age, sex, race, educational achievement, diabetes status, and weight. In our model for change in diastolic BP at 24 month follow-up, weight loss was significantly associated with a reduction in diastolic BP (*p* = 0.03) for all participants and for those in both studies (*p* = 0.02). There were no other significant associations. At 6 and 12 month follow-up, quality of life measures were generally improved, but not significantly. At 24 month follow-up, compared to baseline there was statistically significant improvement in the mental composite score for all participants and for the lifestyle subgroup (*p* < .05). There were no adverse events attributed to the intervention; however, 23 participants had at least one adverse CVD event during follow-up, including: 3 acute myocardial infarctions (1 death), 5 hospital evaluations for angina, 1 coronary artery bypass surgery, 6 episodes of heart failure, 3 hospital evaluations for hypertension, 2 strokes, and 3 others.

## Discussion

In this study to evaluate a diet, PA, and weight loss intervention that promoted a Mediterranean style eating pattern adapted for the southeastern US, participants reported substantial improvements in dietary behaviors (improved consumption of high quality fats and fruit and vegetables) at 6 month follow-up that were generally maintained through 24 months, with increases in self-reported fruit and vegetable consumption corroborated by the objective measure of blood carotenoids at 12 month follow-up. A major difference between the diet pattern tested in this study and most previous dietary intervention trials conducted in the US, including several we have conducted in low-income populations [39, 45–48, 67, 68] was the recommendation to increase consumption of foods containing high quality fats (no restriction on total fat intake), with a focus on increasing fat intake from nuts and vegetable oils. We believe this recommendation to consume more healthful fats was well received because we encouraged consumption of many foods that historically have been an important part of the southern diet, but previously discouraged by dietary guidelines focusing on fat restriction. Perhaps this appealing aspect of the Med-South Diet contributed to the maintenance of dietary change through 24 months.

A major goal of this project was to develop an intervention that would be similarly effective among minority and non-minority participants, in hopes that such an intervention may decrease health disparities. Overall, both lifestyle and physiologic outcomes were similar by race. Moreover, for systolic BP, there were trends towards a greater reduction among African American participants.

Relevant to our findings on BP change, in a sub-study of the PREDIMED randomized trial, Estruch [22] examined the effect on BP of a Mediterranean diet supplemented with extra virgin olive oil (*n* = 257) and nuts (*n* = 257) compared to a low-fat diet (*n* = 256). At 3 month follow-up, systolic BP was reduced by 4.8 mmHg in the olive oil group and 6.5 mmHg in the nuts group, with 0.64 mmHg increase in the low-fat group. In our study, among participants in the lifestyle only group, we observed a similar reduction in systolic BP at 6 and 24 month follow-up, with a smaller reduction at 12 months. Among those taking part in both the lifestyle and high BP studies, the reduction in systolic BP was larger at all follow-up time up, likely due to the combined effect of diet and PA change and improved medication adherence. With regard to blood lipid change, in the PREDIMED sub-study, total cholesterol was reduced by 3.9 mg/dL in the olive oil group and 5.0 mg/dL in the nuts group, while increasing 0.74 mg/dL in the low-fat group. In our study, total cholesterol was reduced 3 mg/dL at 12 and 24 month follow-up. However, in the PREDIMED study, HDL-C increased 2.4 and 0.9 mg/dL in the olive oil and nut groups respectively, while decreasing 0.4 mg/dL in the low-fat group. Despite improved self-reported diet and PA behaviors and modest weight loss, in our study, HDL-C was reduced by 2 and 1 mg/dL, respectively at 12 and 24 month follow-up.

Of the 249 participants taking part in Phase II that returned for 6 month follow-up weights, 231 (93 %) had a BMI > 25 kg/m^2^ and were eligible for the weight loss intervention. It is noteworthy that 138 (60 %) elected to receive the weight loss intervention, suggesting a high level of interest in weight loss among overweight study participants. Though a sub-set of weight loss participants achieved and maintained meaningful weight loss; overall, the weight loss component of this trial did not achieve study goals. First, we expected 60 % of participants to take part in the weight loss program, but only 138 (40 % of enrollees and 55 % of those returning and eligible at 6 month follow-up) elected to do so. Second, for weight loss participants, the average weight loss at the conclusion of the weight loss intervention (baseline to 12 months) of 2.5 kg was less than observed in our previous weight loss intervention trials [40, 41]. Because fewer participants elected to take part in the weight loss intervention and weight loss was less than expected, the embedded RCT of weight loss maintenance was substantially underpowered. Possible reasons for less weight loss than expected include the non-selected sample (in our prior weight loss studies, participants were screened for motivation to lose weight) and an older population with more co-morbidities. An unexpected positive finding was that 20 % of African American participants who returned for follow-up at the conclusion of Phase I, which was not a weight loss intervention, lost ≥ 5 % of body weight.

Though the US Preventive Services Task Force [69] recommends clinicians should offer and refer all adults with BMI ≥ 30 kg/m^2^ to an intensive weight loss program, most published weight loss studies conducted in primary care and community settings [70–72] have included selected samples, often younger, with lower BMI, and with less co-morbidities than those seen in routine practice. In contrast, we offered our weight loss intervention to a non-selected, high risk, older, and largely minority sample. With the caveat that our weight change was compared to baseline and not a control group, our achieved weight loss, though modest, was greater than reported in a recent meta-analysis by Booth [70] assessing behavioral weight loss programs in primary care settings with pooled results across 15 RCTs of −1.4 kg (−2.1 to −0.6). Furthermore, in a recent review [31] of weight loss studies conducted in disadvantaged populations, only 20 % lost more than 5 % of body weight at follow-up, which is less than we achieved in this study. However, overall weight loss was modest, especially at 24 month follow-up, highlighting the need for more effective weight loss interventions for high risk populations, as enrolled in this study.

This study has several limitations. The overall design (excluding the embedded RCT) was a pre-post comparison study with no control group. Thus, observed changes could be due to secular trends or other factors. With regard to secular trends, data on overweight and obesity from the behavioral risk factor surveillance system (BRFSS) for all of North Carolina and for Eastern North Carolina [73] suggest no reduction in weight for the adult population from 2011 to 2014 (see Additional file 2: Table S1). During this same time period, BRFSS data for both North Carolina and eastern North Carolina adults, suggest a slight increase in PA from 2011 to 2014 (see Additional file 3: Table S2). Many outcomes were self-reported, which may be exaggerated due to social desirability reporting bias. Also, we present many comparisons and some *p*-values may be significant by chance. Further, our sample size was relatively small for some of the subgroup outcomes reported. Finally, the generalizability of our findings may be limited to samples similar to those enrolled in this study.

## Conclusions

In this study promoting a Mediterranean style dietary pattern as a major component of a lifestyle and weight loss intervention to reduce CVD risk, the large majority of participants reported substantial improvement in dietary intake and a meaningful percentage lost weight and maintained weight loss. Importantly, as lifestyle and physiologic changes were similar for African American and white participants, this type of culturally tailored intervention has the potential to reduce both CVD risk and disparities in CVD rates. Future research should include RCTs enrolling similar high risk populations that assess change in CVD risk factors and ultimately change in CVD events.

## Abbreviations

BP, blood pressure; BMI, body mass index; CAC, community advisory committee; CVD, cardiovascular disease; dL, deciliter; DRA, dietary risk assessment; HDL-C, high density lipoprotein cholesterol; HHL, heart healthy lenoir; kg, kilogram; PA, physical activity; PREDIMED, prevención con dieta mediterránea; RCT, randomized controlled trial; SES, socioeconomic status; US, United States

## Additional files

Additional file 1: Description of and materials included in the intervention. (PDF 5292 kb) Additional file 2: BRFSS Survey Results for Overweight and Obesity: Eastern North Carolina and North Carolina. (DOCX 14 kb) Additional file 3: BRFSS Survey Results for Exercise: Eastern North Carolina and North Carolina. (DOCX 15 kb)
