# Supplementary material for: A community-based lifestyle and weight loss intervention promoting a Mediterranean-style diet pattern evaluated in the stroke belt of North Carolina: the Heart Healthy Lenoir Project
Source: BMC Public Health. 2016 Aug 5;16:732. doi: 10.1186/s12889-016-3370-9 (PMC4975883; doi:10.1186/s12889-016-3370-9)
Supplement: Additional file 1: — Description of and materials included in the intervention. (PDF 5292 kb) [file 12889_2016_3370_MOESM1_ESM.pdf]

Appendix Table of Contents

Section	Description
1	Phase I: Lifestyle Intervention (Months 1-6)
	a. Description of Intervention and Counseling
	b. Lifestyle Survey
	c. Tip Sheets
	d. Goal Sheets
2	Phase II: Weight Loss Intervention (Months 7-12)
	a. Weekly Group Sessions: Leader's Guide with Session Content
	b. Phone and Group Sessions: Session Content
3	Phase III: Maintenance of Weight Loss Intervention (Months 13-24)

Section 1 Phase I. Lifestyle Intervention (Months 1-6)

Section 1a. Description of Intervention and Counseling

From its inception and when updated, the intervention has been culturally tailored to the dietary patterns of southerners: both the dietary assessment and recommendations focus on foods commonly consumed by southerners, and the cookbook includes many southern style recipes.[1,2] As part of the North Carolina WISEWOMAN (Well-Integrated Screening and Evaluation for WOMen Across the Nation) program evaluation,[3] and in an effort to improve the intervention content and tailor it to the needs of low income North Carolinians, 49 special intervention counselors completed post intervention surveys and provided recommendations for improving the dietary content of the intervention. With a similar focus on cultural relevance, several health department staff who took part in the WISEWOMAN program served on an advisory committee to guide the intervention update undertaken in 2007. In making modifications for the intervention tested in this study, we also included findings from our formative work in Lenoir County.[4] Finally, in developing intervention content consistent with the evolving literature on the importance of high quality fat in the diet,[5] we encouraged consumption of foods that historically have been an important part of the Southern diet, but previously discouraged by healthy eating guidelines focusing on fat restriction, including peanuts; peanut butter;[6] pecans; full fat mayonnaise, salad dressing, and tartar sauce; and frying or sautéing using healthful, but inexpensive and familiar vegetable oils.

The intervention was also adapted from evidence-based lifestyle interventions to reduce CVD risk,[7-10] and retains their theoretical underpinnings, including cognitive and behavioral strategies

shown to effectively promote lifestyle behavior changes,[11-16] such as motivational interviewing, self-monitoring, problem-solving, goal setting, and feedback on goal progress.

At the first session, the lifestyle survey was administered (Section 1c of Appendix), which included a 26-item version of the previously validated Dietary Risk Assessment (DRA)[17,18] and 11 questions assessing physical activity. This was followed by an overview of the program, a review of the major components of a healthy eating plan, and then review of the specific dietary content for Session 1. At the end of the session, the participant and counselor developed an individually tailored action plan to help guide the participant's eating behaviors for the next month (or until the next counseling session). To do so, they first reviewed the DRA assessment page for Session 1 (Section 1c of Appendix, page A-3) and identified current eating behaviors that "could be improved" (middle column) or "need to be improved" (right most column). Then they reviewed the dietary tips for these problematic eating behaviors on the tip sheet for Session 1 (Section 1d of Appendix, page B-24), with tips linked by number and color coding to the items on the DRA. Of note, the tip sheet included recipe suggestions in a "Southern Style" cookbook given to all participants. Finally, the counselor and participant identified 2 achievable goals (participants could opt to choose 1) to work on before the next visit and documented them on the goal sheet (Section 1e of Appendix, page B-28). If time permitted during Session 1, physical activity was also addressed in a similarly tailored way (Section 1c of Appendix, page A-11 for assessment, Section 1d of Appendix, page D-99 for tip sheet, and Section 1c of Appendix, page D-103 for goal sheet) but with only one goal selected. Subsequent sessions followed the same format but opened with a check-in on progress toward goals and addressed diet and physical activity. At the six month follow-up visit, success achieving goals selected at Session 4 was assessed.

REFERENCES

1. Ammerman AS, Haines PS, DeVellis RF, et al. A brief dietary assessment to guide cholesterol reduction in low-income individuals: design and validation. *J Am Diet Assoc.* 1991;91(11):1385-1390.

2. Ammerman AS, DeVellis BM, Haines PS, et al. Nutrition education for cardiovascular disease prevention among low income populations: description and pilot evaluation of a physician-based model. *Patient Educ Couns.* 1992;19(1):5-18.

3. Jilcott SB, Macon ML, Rosamond WD, et al. Implementing the WISEWOMAN program in local health departments: staff attitudes, beliefs, and perceived barriers. *J Womens Health.* 2004;13(5):598-606.

4. Pitts SB, Vu MB, Garcia BA, et al. A community assessment to inform a multilevel intervention to reduce cardiovascular disease risk and risk disparities in a rural community. *Fam Community Health.* 2013;36(2):135-146.

5. Mozaffarian D, Appel LJ, Van Horn L. Components of a cardioprotective diet: new insights. *Circulation.* 2011;123(24):2870-2891.

6. Luu HN, Blot WJ, Xiang YB, et al. Prospective evaluation of the association of nut/peanut consumption with total and cause-specific mortality. *JAMA Intern Med.* 2015;175(5):755-766.

7. Appel LJ, Champagne CM, Harsha DW, et al. Effects of comprehensive lifestyle modification on blood pressure control: main results of the PREMIER clinical trial. *JAMA.* 2003;289(16):2083-2093.

8. Knowler WC, Barrett-Connor E, Fowler SE, et al. Reduction in the incidence of type 2 diabetes with lifestyle intervention or metformin. *N Engl J Med.* 2002;346(6):393-403.

9. Stevens VJ, Obarzanek E, Cook NR, et al. Long-term weight loss and changes in blood pressure: results of the Trials of Hypertension Prevention, phase II. *Ann Intern Med.* 2001;134(1):1-11.

10. Whelton PK, Appel LJ, Espeland MA, et al. Sodium reduction and weight loss in the treatment of hypertension in older persons: a randomized controlled trial of nonpharmacologic interventions in the elderly (TONE). TONE Collaborative Research Group. *JAMA.* 1998;279(11):839-846.

11. Bandura A. *Social Foundations of Thought and Action: A Social Cognitive Theory.* Englewood Cliffs, NJ: Prentice Hall; 1986.

12. Bandura A. Social cognitive theory: an agentic perspective. *Annu Rev Psychol.* 2001;52:1-26.

13. Prochaska J, DiClemente C. Stages and processes of self-change of smoking: toward an integrative mode of change. *J Consult Clin Psychol.* 1983;51(3):390-395.

14. Rollnick S, Mason P, Butler C, eds. *Health Behavior Change: A Guide for Practioners.* London: Churchill Livingstone; 1999.

15. Watson DL. *Self-directed Behavior: Self-modification for Personal Adjustment.* Pacific Grove, CA: Brooks/Cole; 1989.

16. Artinian NT, Fletcher GF, Mozaffarian D, et al. Interventions to promote physical activity and dietary lifestyle changes for cardiovascular risk factor reduction in adults: a scientific statement from the American Heart Association. *Circulation.* 2010;122(4):406-441.

17. Jilcott SB, Keyserling TC, Samuel-Hodge CD, Johnston LF, Gross MD, Ammerman AS. Validation of a brief dietary assessment to guide counseling for cardiovascular disease risk reduction in an underserved population. *J Am Diet Assoc.* 2007;107(2):246-255.

18. Sheridan SL, Draeger LB, Pignone MP, et al. Designing and implementing a comparative effectiveness study of two strategies for delivering high quality CHD prevention: methods and participant characteristics for the Heart to Health study. *Contemp Clin Trials.* 2013;36(2):394-405.

Introduction to the Lifestyle Survey

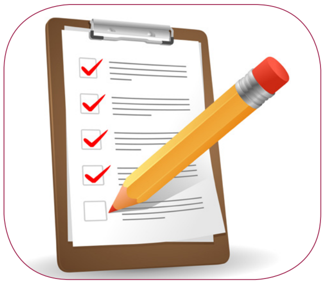

This program is designed to help lower your chances of getting heart disease. To get started, we need to know about your current eating habits.

This survey asks about the kinds of foods you eat. Your answers will help us give you tips on how to lower your chances of getting heart disease.

Instructions

- This survey will take about ten minutes to complete.
- Please choose the answer that best describes what you **USUALLY** do.
- Please pay careful attention to the question and answer choices—some questions will ask you how much you eat in a normal **DAY** and others will ask how much you eat in a normal **WEEK**.
- Remember, your answers to these questions will help us help you.

Eating Patterns Survey

Nuts, Oils, Dressings, and Spreads

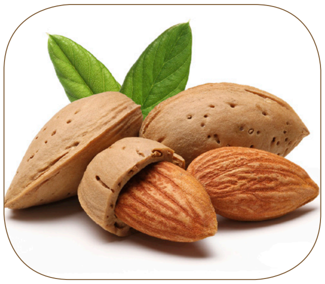

In an average **WEEK**,	Desirable	Could be improved	Needs to be improved
**1.** How many servings of **peanut butter or nuts** (like peanuts, almonds, pecans, walnuts or cashews) do you usually eat?	☐ 3+	☐ 2	☐ 0-1
**2.** What type of **butter** or **margarine** do you usually use?	☐ trans fat free margarine (most tub margarines are trans fat free)	☐ butter	☐ stick margarine

In an average **WEEK**, how many times do you…

**3.** Use **trans fat free margarine** as a topping for bread, biscuits, corn, potatoes, rice, or noodles?	☐ 3+	☐ 1-2	☐ 0
**4a.** Eat **foods fried or sautéed at home using vegetable oil** (olive, soybean, canola, corn oil, or tub/liquid margarine)?	☐ 3+	☐ 1-2	☐ 0
**4b.** Eat food **baked** at **home** using **vegetable oil** or **trans fat free margarine**?	☐ 3+	☐ 1-2	☐ 0
**5.** Use full-fat **salad dressing** (excluding NO-FAT, FAT-FREE, and LOW-FAT dressings)?	☐ 3+	☐ 1-2	☐ 0
**6.** Eat regular **mayonnaise** (excluding NO-FAT, FAT-FREE, or LOW-FAT mayonnaise) on sandwiches or in tuna, chicken, or egg salads?	☐ 3+	☐ 1-2	☐ 0

Eating Patterns Survey

Vegetables, Fruit, Whole Grains, and Beans

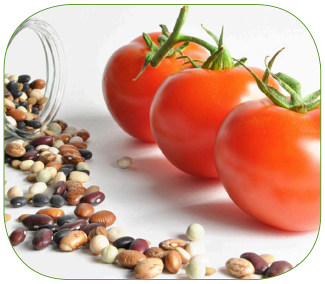

On an average **DAY**, how many servings of these foods do you eat or drink?

	Desirable	Could be improved	Needs to be improved
**1a. Dark-green or orange vegetables** like collard greens, broccoli, tossed salads made with dark-green leafy lettuces, sweet potatoes, butternut squash, or carrots	☐ 2+	☐ 1	☐ 0
**1b. Other vegetables** like corn, green peas, lima beans, okra, zucchini, turnips, onions, cabbage, green beans, or tomatoes (including tomato sauce)	☐ 2+	☐ 1	☐ 0
**2.** Fresh, canned, or frozen **fruit** or 100% fruit juice (½ cup equals a serving)	☐ 3+	☐ 2	☐ 0-1
**3.** Bread, rolls, or tortillas made all or mostly with **white flour**	☐ 0	☐ 1	☐ 2+

In an average **WEEK**, how many servings of these foods do you eat?

**4. Beans or peas** like pinto beans, kidney beans, lentils, or black-eyed peas	☐ 3+	☐ 1-2	☐ 0
**5. White rice or regular pasta**, like noodles, spaghetti, or macaroni	☐ 0	☐ 1-2	☐ 3+
**6. Regular cold or hot cereals**, like sugar-frosted flakes, cocoa cereals, grits, or cream of wheat	☐ 0	☐ 1-2	☐ 3+

Eating Patterns Survey

Drinks, Desserts, Snacks, Eating Out, and Salt

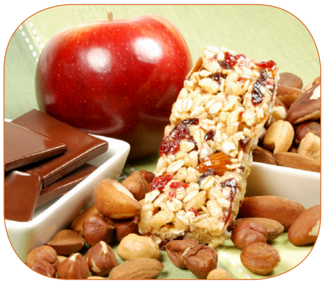

On an average **DAY,** how many 12-oz. servings of sugar-sweetened beverages do you drink with meals or in between meals? One regular can of a beverage is 12 oz.

	Desirable	Could be improved	Needs to be improved
**1.** Sugar-sweetened beverages include regular non-diet sodas, bottled fruit drinks, Kool-Aid, iced or hot coffee or tea sweetened with sugar (including drinks like Coke™, Pepsi™, Sprite™, Snapple™, lemonade, or fruitade and sports or energy drinks)	☐ 0	☐ 1	☐ 2+

In an average **WEEK**, how many servings of these foods do you eat?

**2.** Doughnuts, sweet rolls, pies, cakes, cookies, or candy	☐ 0-1	☐ 2-3	☐ 4+
**3.** Ice cream, ice milk, sherbet, or frozen yogurt	☐ 0-2	☐ 3-4	☐ 5+
**4.** Snack chips (like potato chips, corn chips, tortilla chips, or cheese puffs), crackers, or pretzels	☐ 0-2	☐ 3-4	☐ 5+

In an average **WEEK**...

**5.** How many times do you eat out at **restaurants, get food delivered**, or eat restaurant **carry-out** at home? (Include food from fast-food restaurants.)	☐ 0-2	☐ 3-4	☐ 5+
**6.** When you buy foods like canned soups or beans, snack chips, or crackers, do you usually get products that are **low sodium** or have **no added salt**?	All or most of the time	Sometimes	Rarely/never

Eating Patterns Survey

Fish, Meat, Poultry, Dairy, and Eggs

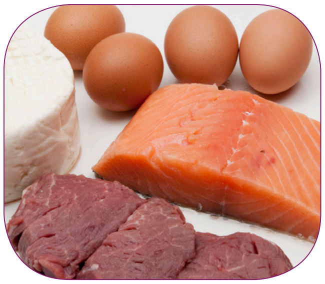

In an average **WEEK**, how many servings of these foods do you eat?	Desirable	Could be improved	Needs to be improved
**1. Fish,** including tuna and shell fish (like shrimp)	☐ 2+	☐ 1	☐ 0
**2. Bacon, sausage, hot dogs** or **cold cuts** like bologna, salami, Spam™ or deli meats including turkey and beef	☐ 0-2	☐ 3-4	☐ 5+
**3. Chicken or turkey,** excluding chicken or turkey cold cuts	☐ 3+	☐ 2	☐ 0-1

On an average **DAY**, how many servings of these foods do you eat?			
**4. Red meat** like roasts, steaks, stew meat, ribs, chops, BBQ, or ham; or hamburger, either alone or in dishes like meatloaf and spaghetti sauce	☐ 0-1	☐ 2	☐ 3+

On average, how many servings of these foods do you eat?	
**5. Dairy products**, such as milk, cheese, and yogurt	Number of servings per day _____ or Number of servings per week _____
**6. Eggs**, including in cooking?	Number of servings per day _____ or Number of servings per week _____

Lifestyle Survey

Walking

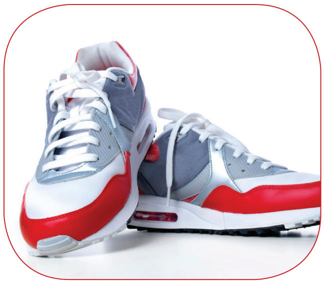

In a usual WEEK…

**1.** How many times do you **walk** for recreation, health, fitness, or transportation such as walking around the block, walking your dog, or walking to work? (**Do NOT** include walking that you do as part of your job.)	☐ 5+	☐ 3-4	☐ 1-2<br>☐ 0<br>(If 0, skip questions 2-5)

On a typical DAY when you WALK for recreation, health, fitness, or transportation…

**2.** What is the **total time** you spend **walking**?	☐ 30 or more minutes	☐ 10-19 minutes<br>☐ 20-29 minutes	☐ Less than 10 minutes
**3.** What is your usual **speed**?	☐ Fairly fast (3-4 miles in an hour)<br>☐ Very fast (more than 4 miles in an hour)	☐ Average or normal (2-3 miles in an hour)	☐ Casual strolling or walking (less than 2 miles in an hour)

On a typical DAY at work…

**4.** What is the **total time** you spend **walking**?	☐ 30 or more minutes	☐ 10-19 minutes<br>☐ 20-29 minutes	☐ Less than 10 minutes<br>☐ Do not walk at work or not employed (skip next question)
**5.** What is your usual **speed**?	☐ Fairly fast (3-4 miles in an hour)<br>☐ Very fast (more than 4 miles in an hour)	☐ Average or normal (2-3 miles in an hour)	☐ Casual strolling or walking (less than 2 miles in an hour)

Lifestyle Survey

Other Types Of Physical Activity

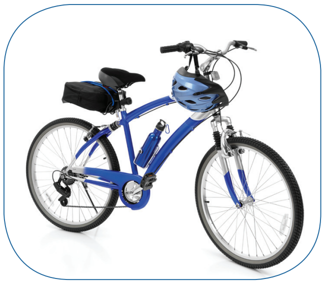

The next questions are about **leisure time** physical activity other than walking. When answering these questions, **DO NOT** include walking. These questions ask about two levels of physical activity: **moderate** and **vigorous**.

MODERATE physical activities cause a moderate increase in breathing and heart rate. **You should be able to carry on a conversation when doing these activities.**

VIGOROUS physical activities cause a large increase in breathing and heart rate. **It is difficult to talk when doing these activities.**

In a usual **WEEK**…

**1.** How many times do you do **moderate** leisure time physical activities like dancing, cycling, social tennis, golf, or gardening?	☐ 5+	☐ 3-4	☐ 1-2<br>☐ 0 (If 0, skip to question 3)

On a typical **DAY** when you do **MODERATE ACTIVITY**…

**2.** What is the **total time** you spend doing this activity?	☐ 30 or more minutes	☐ 10-19 minutes<br>☐ 20-29 minutes	☐ Less than 10 minutes

In a usual **WEEK**…

**3.** How many times do you do **vigorous** leisure time physical activities like jogging, aerobics, swimming laps, or competitive tennis?	☐ 5+	☐ 3-4	☐ 1-2<br>☐ 0 (If 0, skip to question 5)

Lifestyle Survey

Other Types Of Physical Activity

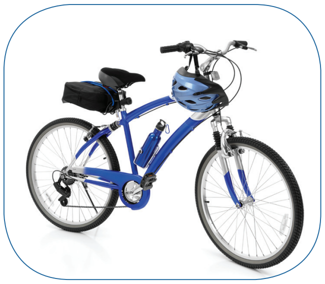

On a typical **DAY** when you do **VIGOROUS ACTIVITY**…

**4.** What is the **total time** you spend?	☐ 20 or more minutes	☐ 10-19 minutes	☐ Less than 10 minutes

5. On a typical **WORK DAY** do you do **MODERATE** or **VIGOROUS ACTIVITY** other than walking?

☐ yes

☐ no (done with survey)

☐ not employed (done with survey)

**6.** What is the **total time** you spend doing this type of activity each day?	☐ 30 or more minutes	☐ 10-19 minutes<br>☐ 20-29 minutes	☐ Less than 10 minutes

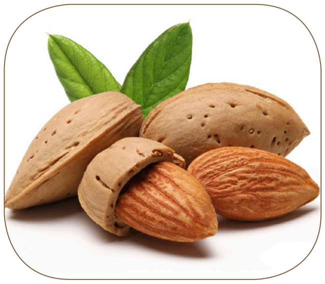

Nuts, Oils, Dressings, and Spreads | Tips

Check the goals you want to work on for the next contact.

☐ **1. Choose nuts and nut butters often. Try to eat three or more servings each week.**

- Enjoy a variety of nuts (pecans, peanuts, walnuts, almonds, cashews) and nut butters, like peanut butter.
- A serving is a handful of nuts (one quarter cup or about one ounce) or two tablespoons of nut butter (about the size of a ping-pong ball).
- Try a peanut butter sandwich on whole grain bread, or peanut butter on celery or an apple. These can make a tasty lunch or snack!
- Unsalted or lightly salted nuts or all natural nut butters are good choices, but regular nuts and nut butters are also good choices.

☐ **2. Choose trans fat free margarine.**

- For spreads, trans fat free margarine is preferred because it contains healthy vegetable oil, but butter on occasion is okay.
- When you use margarine look for tub or squeeze margarines that say trans fat free.
- Don't use regular stick margarines, which are usually high in trans fats.

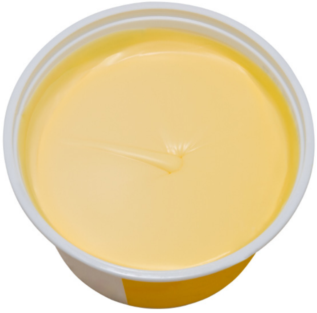

☐ **3. Use healthful toppings like trans fat free margarine or vegetable oil for bread and other foods. Aim for three or more servings per week.**

- Margarine that does not contain trans fat is a healthful choice for topping bread and other foods.
- Consider dipping bread in olive oil or a mix of olive oil and balsamic vinegar.

Continued ➔

Tips

Nuts, Oils, Dressings, and Spreads

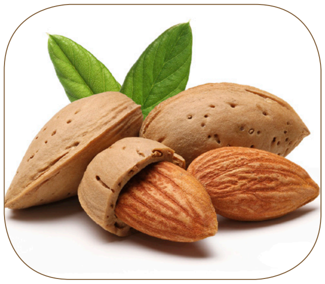

☐ 4. Use healthy oils for frying, sautéing, and baking.

- Use the following oils for sautéing, frying, cooking, and baking: olive, canola, corn, peanut, and soybean oil. Also, some stores sell "vegetable oil," which is a mix of these oils and is also a good choice.
- When frying, sautéing, or baking, use the right oil for the job. Olive oil or canola oil are best for everyday sautéing or frying at low to medium high temperatures. For high heat (to stir fry or deep fry), use peanut, soybean, or corn oil.

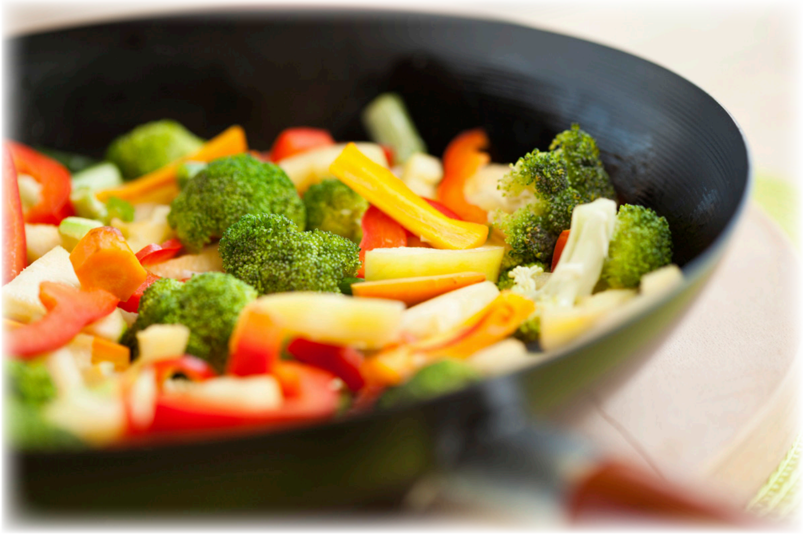

- For everyday cooking, use oils that don't cost too much. Save extra virgin olive oil for salad dressing and dipping. Use other vegetable oils for frying, sautéing, and baking.

☐ 5. Choose healthy salad dressings. Aim for three or more servings per week.

- For store-bought, use regular rather than low-fat or fat-free dressings. Regular store-bought salad dressings contain the healthy types of vegetable oil. Fat-free dressings are often higher in sodium and sugar.
- Consider making your own salad dressing (see Cookbook, pages 98-100).
- Try to select salad dressings with less than 350 mg of sodium per serving. See pages C-81 and C-82 for tips on reading food labels.

☐ 6. Store-bought mayonnaise has fats that are good for your heart. Aim for three or more servings per week.

- Use regular store-bought mayonnaise. It is usually made with soybean or canola oil, both good oils for your heart.
- A serving of mayonnaise is about one tablespoon.

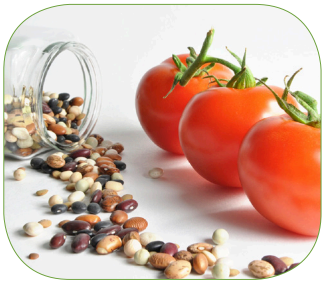

Vegetables, Fruit, Whole Grains, and Beans

Tips

Check the goals you want to work on for the next contact.

☐ 1. Try for four or more servings of vegetables each day.

A word about serving sizes:

- In general, a half cup of a cooked vegetable and one cup of a raw vegetable (salad) is a serving size.
- Though it is good to aim for about four or more servings a day, it is also important to eat a variety of vegetables, as noted below.

Eat a variety of dark-green and orange vegetables.

- Try broccoli or greens.
- Include spinach, romaine, or other dark-green leafy lettuce in salads.
- Add chunks of sweet potatoes, butternut squash, or carrots to steamed or stir-fried vegetables.

Keep costs down.

- Buy fresh vegetables in season (look for a farmer's market).
- Buy seasonal vegetables in bulk and freeze or can some for later.
- Grow your own.

Go easy on starchy vegetables like white potatoes, corn, green peas, and lima beans.

- Try to eat no more than one or two starchy vegetables a day.
- Eat regular potatoes in moderation, either baked, boiled, or fried in vegetable oil.
- Try a baked sweet potato as a substitute for a baked white potato.
- Add other vegetables to potatoes. Bake a vegetable dish with sliced potatoes, carrots, and green beans drizzled with vegetable oil and herbs or other low-salt seasonings (see Cookbook, pages 43-55).

Tips

Vegetables, Fruit, Whole Grains, and Beans

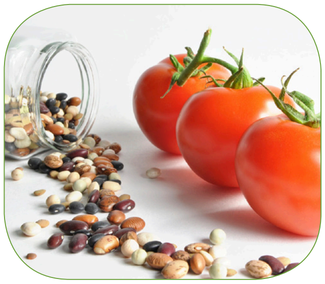

Add variety to meals with vegetables.

- Add onions, mushrooms, peppers, tomatoes, or salsa to your omelets.
- Make sandwiches, wraps, or burritos with lots of lettuce, tomatoes, carrots, onions, or other sliced vegetables.

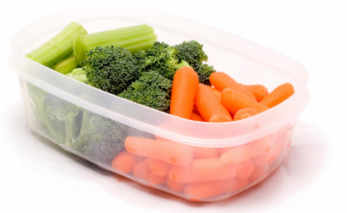

- Bring cut-up vegetables to work instead of eating vending machine snacks.
- Steam, fry, or sauté vegetables in vegetable oil.
- Buy frozen/canned vegetables or canned beans and peas that have low- or reduced salt (sodium). Rinse canned vegetables that have added salt. See pages C-77 and C-78 for tips on adding flavor without adding salt.

Spice up your vegetables

- Use onions, garlic, vinegar, herbs, spices, vegetable oil, or trans fat free margarine to season and add flavor to fresh greens and other vegetables. (See "Fresh Greens, Southern Style" and other ways of preparing vegetables on pages 43-55 of the Cookbook.)
- Use broccoli, carrots, or squash in place of white rice or pasta in your favorite soup or casserole.

☐ 2. Try for three or more different fruits each day.

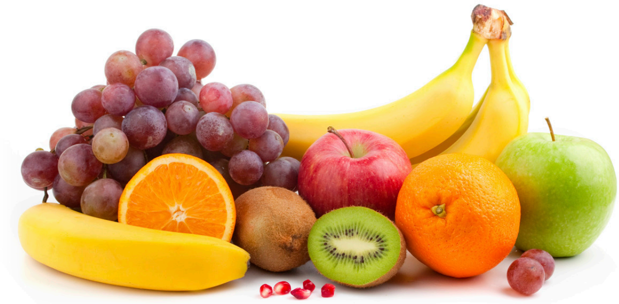

- Fruit makes a tasty snack or dessert.
- Eating more fruit doesn't have to be expensive; buy fruit that is in season or pick your own.
- Add fruits like bananas, berries, or peaches to your cereal.
- Use very ripe fruit to make a blender smoothie (see pages 87-91 of the Cookbook for other fruit recipes).
- Dried fruit such as raisins, cranberries, or apricots are also good snacks but being mindful of portion size is important. One quarter of a cup or about a handful is considered one serving.
- As you add more fruit, make sure to do so in the healthiest ways.
- Eat whole fruit (fresh, frozen, or canned) instead of drinking fruit juice—whole fruit gives you more fiber, which also can help you feel full.
- Avoid canned or frozen fruits with added sugar or syrup (look for canned fruit labeled "in its own juice").
- If you do get canned fruit in light or heavy syrup, drain off the syrup and eat the fruit without it.

Continued ➔

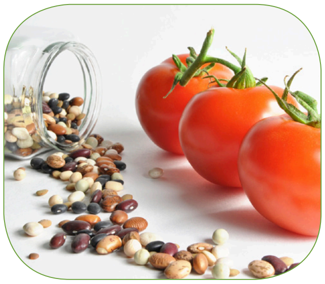

Vegetables, Fruit, Whole Grains, and Beans | Tips

3. Choose more whole grain breads. Try to eat two or more servings of whole grain bread products each day.

- Pick whole grain bread instead of white bread. Remember that multi-grain is not the same as whole grain. Look for whole wheat flour or whole grains as the first ingredient on the food label.
- Check the label and choose breads with at least two grams of fiber per slice.

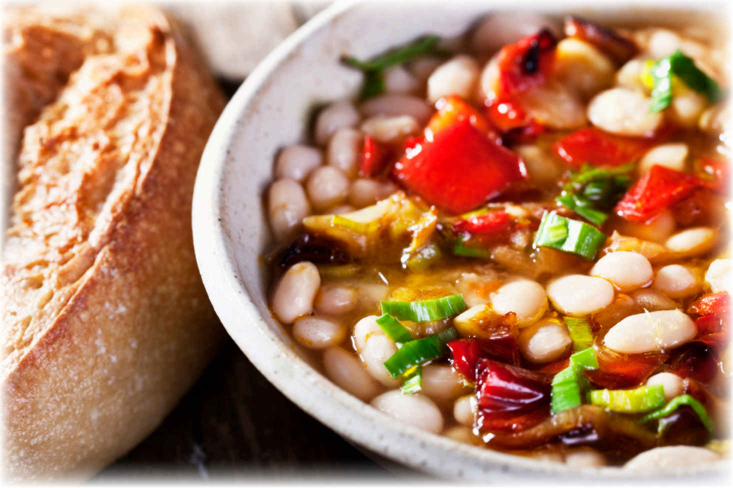

4. Eat more beans and peas. Try to eat beans or peas three or more times per week.

- Beans and peas are a good source of fiber and protein and therefore can be a healthy, high fiber substitution for meat. They are also less expensive than meat.
- Soak beans overnight to shorten the cooking time.
- Use onions and garlic for seasoning, or season with vegetable oil or small pieces of ham, turkey, or beef.
- Make a quick healthy meal with canned beans. Get the low-salt (low sodium) kind, or drain and rinse canned beans. See pages C-79 and C-80 for more information on cooking for one or on the run.

5. Try other whole grain foods like brown rice, barley, and whole grain pasta. Aim to eat three or more servings each week.

- Whole grains are foods like brown rice and whole wheat bread and pasta. These foods are high in fiber and B vitamins.
- Though brown rice tastes a bit different from white rice, it's easy to get used to the taste. Many people prefer the taste of brown rice.
- To get used to the taste of whole grain pasta, try mixing with regular pasta at first.

Tips

Vegetables, Fruit, Whole Grains, and Beans

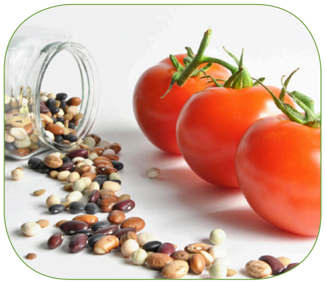

☐ 6. Eat whole grains for breakfast. Try for three or more servings per week.

- Choose whole grain cereals (ready-to-eat or cooked). Cereals like shredded wheat, bran flakes, or old-fashioned oatmeal are all good choices.
- Stay away from cereals with lots of added sugar (like frosted flakes). Aim for three grams of sugar per serving or less. (See pages C-81 and C-82 for more information about how to read labels.) If your cereal has dried fruit such as raisins aim for five grams of sugar per serving or less. You can sweeten your cereal by topping with bananas, berries, peaches, or other fruit.
- Munch on whole grain cereals for a healthy snack any time of the day!
- Avoid instant grits and other cereals that are high in salt (sodium). Aim for no more than 350 mg of salt per serving.

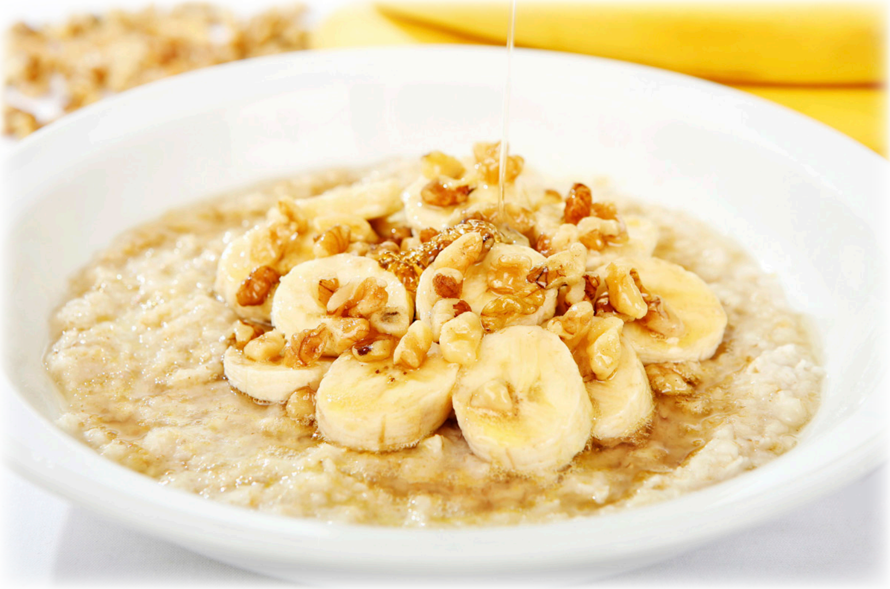

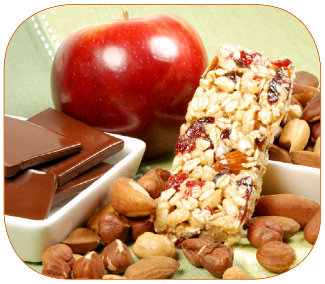

Drinks, Desserts, Snacks, Eating Out, and Salt

Tips

Check the goals you want to work on for the next contact.

☐ 1. Choose healthy drinks.

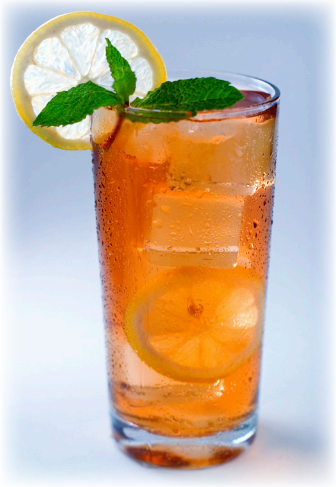

- Aim to replace sugar-sweetened drinks with healthy drinks like water, lightly sweetened tea or coffee, milk, or 100% fruit juice.
- Stay away from non-diet sodas, bottled fruit drinks (such as fruit punch or fruit-aides), sports drinks, energy drinks, and other sugary beverages—they are full of sugar, which means they provide a lot of calories but are not filling.
- Avoid drinks that list sugar as one of the first ingredients. There are many different types of sugar. Try to avoid sweeteners like high fructose corn syrup (and other syrups), sucrose, malt, fructose, or honey.
- Good choices are unsweetened or lightly sweetened iced tea, hot tea, or coffee. If you need to sweeten your tea or coffee, one to two teaspoons per cup or glass is okay (so is a little cream, like one to two tablespoons of half-and-half). This amount of sugar is much better than the nine teaspoons in a 12-ounce soda.
- Other good choices are:
  - Seltzer water with a touch of lemon, lime, or fruit juice.
  - 100% fruit juice, but aim for one cup or less per day.
  - Milk.
  - Diet soda.

☐ 2. Watch out for sweets. Choose other foods for snacks or desserts.

- Fill up on healthy foods so you are not as hungry for sweets.
- Try fruit for dessert.
- Save sweets for special occasions (birthdays and holidays), not every day.
- When you do eat sweets, eat small amounts.

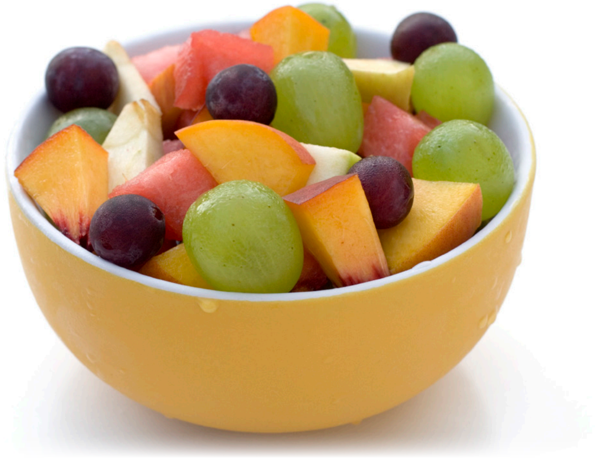

Tips

Drinks, Desserts, Snacks, Eating Out, and Salt

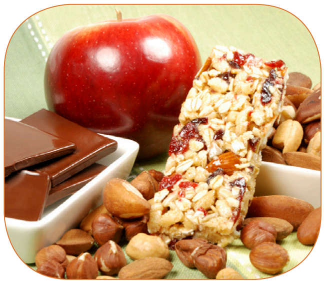

- Stay away from store-bought doughnuts, sweet rolls, pies, cakes, cookies, candy bars (except dark chocolate), milk chocolate, caramel candies, and cream-filled desserts. They are often high in sugar and may have trans fats.
- A small amount of dark chocolate (more than 50% cocoa solids) is a good choice for dessert. Dark chocolate in small amounts (half to one ounce per serving) is thought to be good for your heart.

☐ 3. Choose cold and frozen desserts with care. Aim to eat two or fewer servings of frozen dessert each week.

- For a refreshing summer dessert, have a chilled slice of watermelon or a bowl of fresh berries with vanilla yogurt.
- Try popsicles as a tasty treat. Be sure store-bought popsicles are made with 100% fruit juice, or make your own. Freeze 100% fruit juice in small cups with popsicle sticks.
- If you want a frozen dessert, eat a small amount (about half a cup) of ice cream, ice milk, fruit ice, sherbet, or frozen yogurt.

☐ 4. Be careful with snack chips and crackers.

- Choose lightly salted chips and crackers made with vegetable oil.
- Check the ingredients list of snack chip packages and avoid items that contain partially hydrogenated vegetable oil or trans fat on the food label.
- Try chips or crackers made with vegetable oil.
- When possible, choose whole grain crackers with no trans fats.
- Instead of snack chips or crackers, try raw vegetables, fruit, or a small handful of nuts. These are healthier options.

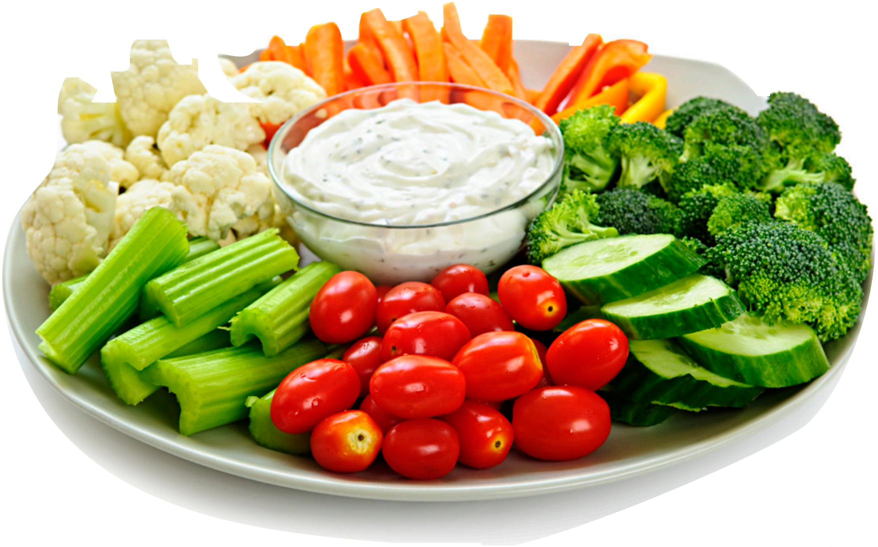

Continued →

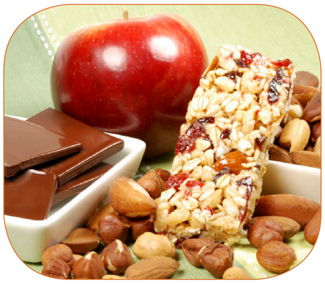

Drinks, Desserts, Snacks, Eating Out, and Salt

Tips

5. Make good choices when you eat at restaurants.

- When you eat out, choose what you eat carefully.
- Choose healthy menu items such as:
  - Grilled or baked red meat, poultry or fish or fried poultry or fish if you know that non-trans fat oil is used.
  - Tossed vegetable salads with one to two tablespoons of dressing.
  - Steamed or sautéed vegetables.
  - Fruit plates.
  - Whole grain breads.
  - Beans.
  - Water with a twist of lemon.

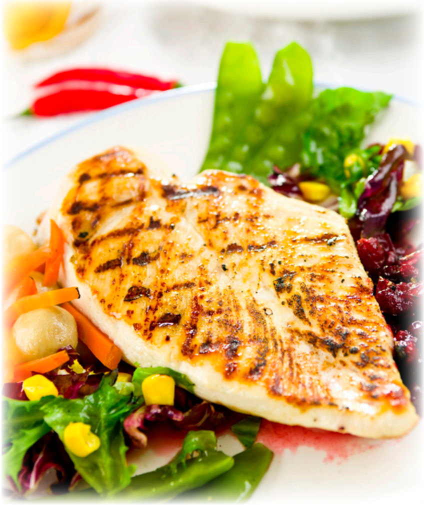

- Fried foods are okay if fried in vegetable oil without trans fats.
- Preparing food at home is a good option because you know what ingredients are used. This is especially true for fried foods because sometimes it is not easy to find out if restaurants use healthy oils.
- Avoid drinks with lots of sugar. In restaurants that have unsweetened and sweetened tea, ask for a mixture (half unsweetened and half sweetened).
- As portions sizes are often large, split an order with a friend, or eat half of the portion and take the rest home for another meal.
- Order small portion sizes.

6. Go the low-salt way. Try to buy low sodium or no added salt foods all or most of the time.

- Most of the salt that people eat comes from restaurant or packaged foods.
- Buy foods that say low- or reduced salt (sodium) or no salt added.
- Eat out less often in restaurants—even healthy restaurant meals often have a lot of salt.
- Use seasonings like pepper, lemon juice, vinegar, herbs, and spices. See pages C-77 and C-78 for suggestions on adding flavor to your food without adding salt. Mix your favorite herbs or spices in a handy shaker.
- Watch out for packaged or frozen meals with noodles. These can be high in salt and trans fats. See pages C-81 and C-82 for tips on reading food labels.

Tips

Fish, Meat, Poultry, Dairy, and Eggs

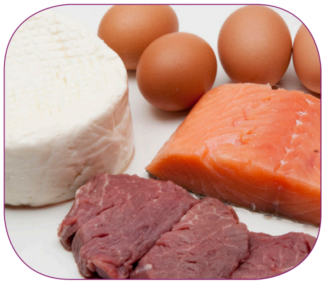

Check the goals you want to work on for the next contact.

☐ 1. Choose fish more often. Aim for one or more servings of fish per week.

- Try baked, broiled, grilled, or fried fish. See Cookbook, pages 19-25 for tasty ways to cook fish.
- When frying fish, use vegetable oils instead of shortening or lard.
- Try some of the fish that have healthy fats, like canned tuna, herring, canned or fresh salmon, or lake trout.
- When you eat canned tuna, choose light tuna (most albacore or white tuna is higher in mercury).

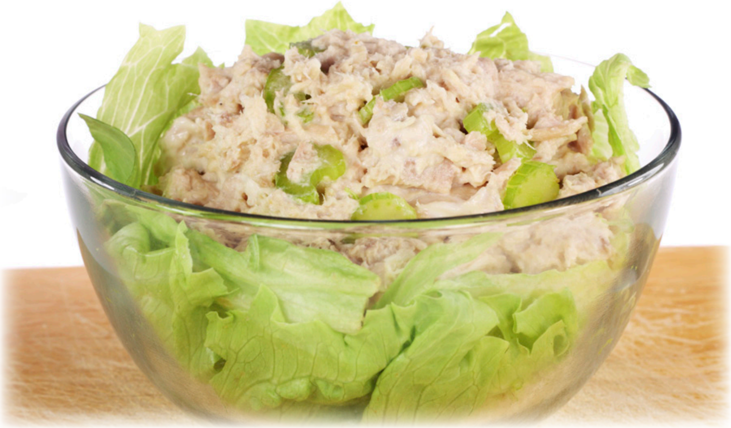

- Some fish contain mercury, which can be harmful if large amounts are eaten. So eat fish with low levels of mercury, such as salmon, catfish, shrimp, and canned light tuna.

☐ 2. Cut down on bacon, sausage, hot dogs, and cold cuts. Aim for three times a week or less.

- Try having breakfast without meat on some days.
- Try sandwiches made with:
  - Sliced fresh beef, pork, chicken, or turkey as fresh meat are better choices than processed meats (cold cuts).
  - Chicken, egg, or tuna salad (these "salad" sandwiches usually contain mayonnaise, which has healthy fats that are good for your heart).
  - Peanut butter and jelly or banana.
- Also, try these other ideas for lunches:
  - Healthy leftovers.
  - Soups (low salt/sodium).
  - Salads with dressing.

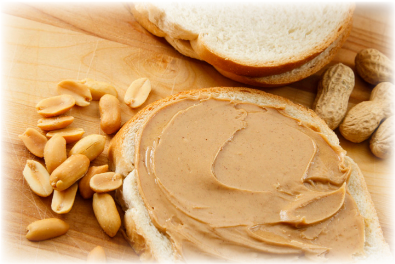

Continued ➔

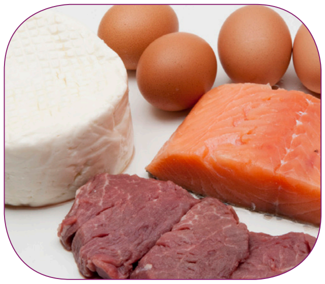

Fish, Meat, Poultry, Dairy, and Eggs

Tips

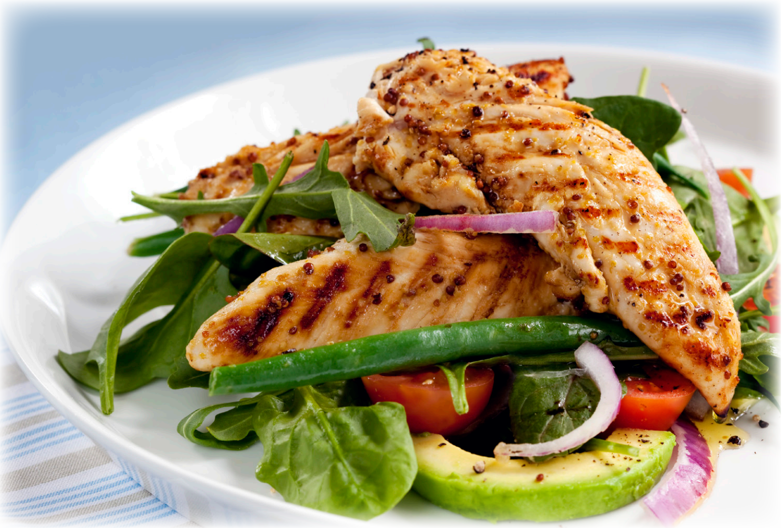

☐ **3. Eat chicken and turkey often. It is reasonable to eat three or more servings of poultry each week.**

- Bake, broil, or barbecue chicken. See recipe for crispy baked chicken on page 16 in our Cookbook.
- Fried chicken is also okay, if fried in healthy vegetable oils.
- Do not fry chicken in shortening as most shortening still has trans fats.
- Fresh chicken or turkey is better for sandwiches than chicken or turkey cold cuts.

☐ **4. If you like eating red meat, like beef, pork, or lamb, aim for no more than one serving per day.**

- Red meat is high in saturated fat. Although experts no longer think saturated fat causes heart disease, it does not reduce the risk of heart disease like some other foods. Also, high intake of red meat may lead to a modest increase in colon cancer. Up to one serving per day is a good goal.

☐ **5. Dairy products, like milk cheese and yogurt, are good choices.**

- Full fat dairy products are high in saturated fat, but do not seem to increase the risk for heart disease. If you enjoy dairy products, two to three servings per day of low- or full fat dairy products is a good goal.
- Dessert dairy products, like ice cream, ice milk, and frozen yogurt are high in sugars and it is best to save these treats for a couple of times a week.

☐ **6. Eggs are also a good food choice.**

- It is true that eggs are high in cholesterol and in the past there were recommendations to lower egg intake. However, eggs are also very high in other nutrients and egg intake of one to two per day does not increase the risk of heart disease. If you enjoy eggs, eating one to two a day is fine.

Tips

Walking Steps

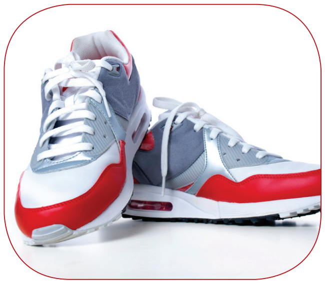

✔ Check the goal you want to work on based on the number of steps you take each day. Then, review the tips for reaching your goals on the next page.

Current Steps per Day	✔	Goals for Next Contact (check the box to indicate your goals)	Long-Term Goal
Less than 5,000		Increase steps by **500 per day** (about five minutes of walking); after two weeks increase by another 500 per day. In one month, goal is **1000 more steps per day**.	7,500 or more steps per day
		Increase steps by **500 per day** (about five minutes of walking); after each week, increase by another 500 per day. In one month, goal is **2000 more steps per day**.	
5,000-7,499		Increase steps by **500 per day** (about five minutes of walking); after two weeks increase by another 500 per day. In one month, goal is **1000 more steps per day**.	10,000 or more steps per day
		Increase steps by **1000 per day** (about ten minutes of walking); after two weeks increase by another 1000 per day. In one month, goal is **2000 more steps per day**.	
7,500-9,999		Increase steps by **500 per day** (about five minutes of walking); after two weeks increase by another 500 per day. In one month, goal is **1000 more steps per day**.	10,000 or more steps per day
		Increase steps by **1000 per day** (about ten minutes of walking); after two weeks increase by another 1000 per day. In one month, goal is **2000 more steps per day**.	
10,000-12,499		Maintain your steps at this level.	Maintain. More activity may be better.
		More activity is better. Aim for 12,500 steps on most days.	
12,500 or more		Maintain your steps at this level.	Maintain.
		Maintain at this level and add muscle-strengthening activities twice a week.	

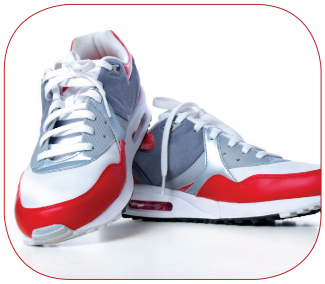

Walking Steps

Tips for Walking More Often and Meeting Your Goals for Steps:

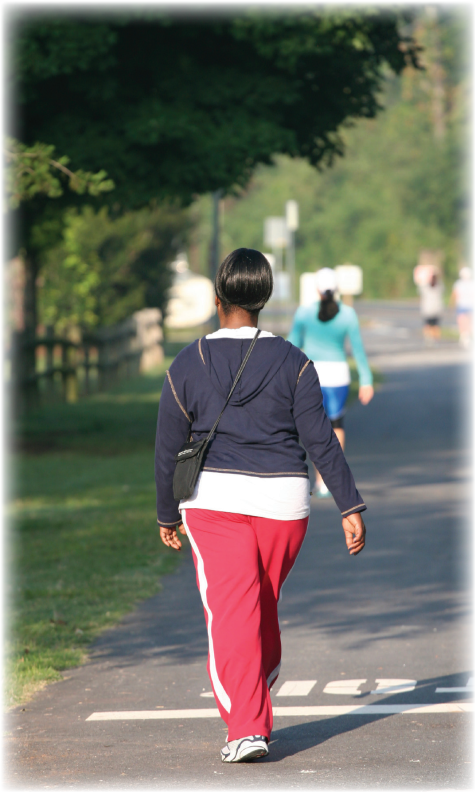

- Try to make walking something you do every day.
- You can walk for recreation, fitness, or for transportation.
- Walk with friends or family or a pet. It'll be healthy for you and for them!
- Look for opportunities to walk during every day activities… try for ten or more minutes at a time.
- Build up slowly. Use a pedometer to track your steps and meet your step goals.
- Walking fast has health benefits. Aim for three to four miles per hour or fast enough to increase your heart rate and breathing.

Tips for Muscle-Strengthening Activities:

- Pick exercises that work for you: stretch bands, chair exercises, weights, and working against gravity are all good activities. See pages E-133 to E-148 for instructions on how to get started with each type of muscle-strengthening activity.
- Start with 8-12 repetitions or one set of each activity. Work up to two or three sets per muscle group.
- Select activities that work the large muscles in your arms, legs, and mid-section.

Tips

Walking Minutes

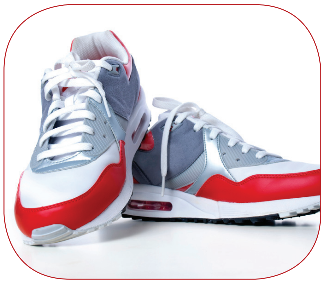

✔ Check the goal you want to work on based on minutes of activity each day. Then, review tips for reaching your goals below.

Current Level of Walking	Goals for Next Contact (check the box to indicate your goals)		Long-Term Goal
Less than 30 minutes five times a week		Walk more often. See tips for walking more, below.	30 minutes on most days of the week.
30 minutes five times per week or more		Maintain this level of physical activity.	Maintain.
		Maintain at this level and add muscle-strengthening activities twice a week. See tips for muscle-strengthening activity below.	Maintain.

Tips for Walking More Often and Meeting Your Goals for Minutes:

- Try to make walking something you do every day.
- You can walk for recreation, fitness, or for transportation.
- Walk with friends or family or a pet. It'll be healthy for you and for them!
- Look for opportunities to walk during everyday activities…try for ten or more minutes at a time.
- Build up slowly.
- Walking fast has health benefits. Aim for three to four miles per hour or fast enough to increase your heart rate and breathing.

Tips for Muscle-Strengthening Activities:

- Pick exercises that work for you: stretch bands, chair exercises, weights, and working against gravity are all good activities. See pages E-133 to E-148 for instructions on how to get started with each type of muscle-strengthening activity.
- Start with 8-12 repetitions or one set of each activity. Work up to two or three sets per muscle group.
- Select activities that work the large muscles in your arms, legs, and mid-section.

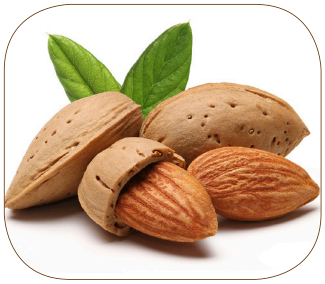

Nuts, Oils, Dressings, and Spreads

My Goals and First Steps

Date: ____________________

Goal 1: ____________________________

____

____

Goal 2: ____________________________

____

____

First Steps: ____________________________

____

____

First Steps: ____________________________

____

____

Place this sheet where you will see it often (refrigerator or bathroom mirror). You may monitor goals by the week (left side of table) or by the day (right side of table).

GOAL 1: Did you meet your goal? (Circle ☺ for Yes, or ☹ for No)

Week	Weekly Goal	Daily Goal						
		Monday	Tuesday	Wednesday	Thursday	Friday	Saturday	Sunday
1	☺ ☹	☺ ☹	☺ ☹	☺ ☹	☺ ☹	☺ ☹	☺ ☹	☺ ☹
2	☺ ☹	☺ ☹	☺ ☹	☺ ☹	☺ ☹	☺ ☹	☺ ☹	☺ ☹
3	☺ ☹	☺ ☹	☺ ☹	☺ ☹	☺ ☹	☺ ☹	☺ ☹	☺ ☹
4	☺ ☹	☺ ☹	☺ ☹	☺ ☹	☺ ☹	☺ ☹	☺ ☹	☺ ☹
5	☺ ☹	☺ ☹	☺ ☹	☺ ☹	☺ ☹	☺ ☹	☺ ☹	☺ ☹

GOAL 2: Did you meet your goal? (Circle ☺ for Yes, or ☹ for No)

Week	Weekly Goal	Daily Goal						
		Monday	Tuesday	Wednesday	Thursday	Friday	Saturday	Sunday
1	☺ ☹	☺ ☹	☺ ☹	☺ ☹	☺ ☹	☺ ☹	☺ ☹	☺ ☹
2	☺ ☹	☺ ☹	☺ ☹	☺ ☹	☺ ☹	☺ ☹	☺ ☹	☺ ☹
3	☺ ☹	☺ ☹	☺ ☹	☺ ☹	☺ ☹	☺ ☹	☺ ☹	☺ ☹
4	☺ ☹	☺ ☹	☺ ☹	☺ ☹	☺ ☹	☺ ☹	☺ ☹	☺ ☹
5	☺ ☹	☺ ☹	☺ ☹	☺ ☹	☺ ☹	☺ ☹	☺ ☹	☺ ☹

My Goal and First Steps

Walking

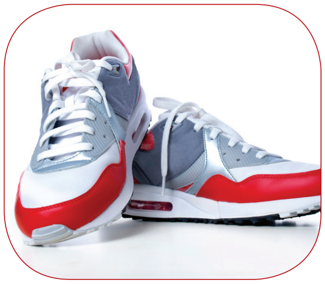

Date: ____________________

Physical Activity Goal:

Steps per day:____________________

Or

Minutes per day:__________________

First Steps to Achieve Your Goal: ____

____

____

Place this sheet where you will see it often (refrigerator or bathroom mirror). You may monitor goals by the week (left side of table) or by the day (right side of table).

GOAL 1: Did you meet your goal? (Circle ☺ for Yes, or ☹ for No)

Week	Weekly Goal	Daily Goal						
		Monday	Tuesday	Wednesday	Thursday	Friday	Saturday	Sunday
1	☺ ☹	☺ ☹	☺ ☹	☺ ☹	☺ ☹	☺ ☹	☺ ☹	☺ ☹
2	☺ ☹	☺ ☹	☺ ☹	☺ ☹	☺ ☹	☺ ☹	☺ ☹	☺ ☹
3	☺ ☹	☺ ☹	☺ ☹	☺ ☹	☺ ☹	☺ ☹	☺ ☹	☺ ☹
4	☺ ☹	☺ ☹	☺ ☹	☺ ☹	☺ ☹	☺ ☹	☺ ☹	☺ ☹
5	☺ ☹	☺ ☹	☺ ☹	☺ ☹	☺ ☹	☺ ☹	☺ ☹	☺ ☹

Section 2 Phase II. Weight Loss Intervention (Months 7-12)

Weight Loss Intervention (Group Only): Leader's Guide Session Content

Session	Session Content
1	▪ Welcome participants to the program, discuss goals and establish expectations<br>▪ Encourage success by outlining reward system<br>▪ Explain and discuss weight loss mechanism<br>▪ Facilitate and encourage getting started with exercise and self-monitoring<br>▪ Review and discuss the use of the Calorie Counter and Food and Fitness Diary
2	▪ Help participants complete the self-monitoring data summary forms and overcome challenges associated with keeping a daily record<br>▪ Help participants identify physical activities that are easy to do and emphasize the importance of all types of movement, the more the better<br>▪ Help participants distinguish between tiredness and more serious issues when exercising
3	▪ Begin to focus on the fruit and vegetable components as part of a diet<br>▪ Help participants set reasonable short-term goals<br>▪ Help participants become aware of the foods that have the most impact on their total calories
4	▪ Evaluate progress on action plans<br>▪ Provide alternatives to typical high calorie meals<br>▪ Discuss the importance of stretching and building muscle
5	▪ Distribute and discuss personalized feedback graphs<br>▪ Discuss and practice designing healthy breakfast meals
6	▪ Heighten awareness of making time for important things in life<br>▪ Develop understanding of unhealthy breakfast patterns
7	▪ Increase skills at purchasing foods to help meet healthy lifestyle and weight loss goals<br>▪ Try new lunch<br>▪ Learn how to plan ahead
8	▪ Discuss what stress is and how to identify stress levels<br>▪ Explore strategies for reducing stress<br>▪ Review portion control as a method of calorie reduction

**9**	▪ Increase skills at preparing foods to help meet healthy lifestyle and weight loss goals<br>▪ Try new dinner ideas<br>▪ Learn how to adapt or modify recipes for healthier eating
**10**	▪ Increase skills at recognizing disordered eating patterns that contribute to overeating<br>▪ Try new snack ideas<br>▪ Learn how to plan ahead for healthier snacking
**11**	▪ Learn how to modify or adjust meals when dining out<br>▪ Discuss strategies for dining out<br>▪ Identify strategies for lowering calories at restaurants
**12**	▪ Review progress to date and discuss challenges/barriers<br>▪ Increase understanding of both helpful and harmful ways in which family can affect weight loss efforts<br>▪ Improve ability to communicate with family about weight loss needs
**13**	▪ Recognize that everyone has negative thoughts and be able to identify examples of them<br>▪ Learn how to stop negative thoughts and replace them with positive ones<br>▪ Explore negative emotional states and discuss how negative emotions may disrupt efforts to maintain a healthy diet and regular physical activity<br>▪ Learn to identify distortions present in negative thoughts<br>▪ Develop affirmations to say to themselves during the difficult times
**14**	▪ Participate in an exercise activity<br>▪ Discuss characteristics of people who successfully lost weight<br>▪ Review their own self management technique<br>▪ Determine calorie needs for maintenance<br>▪ Identify high-risk situations that could lead to return of old habits and create a plan for preventing relapse<br>▪ Create personal rules or boundaries for eating while maintaining weight
**15**	▪ Practice choosing foods wisely<br>▪ Discuss how to stay motivated<br>▪ Plan for special occasions<br>▪ Review ways to continue exercise
**16**	▪ Celebrate accomplishments<br>▪ Discuss feelings associated with moving on to the next phase<br>▪ Discuss and plan for ongoing social support<br>▪ Discuss importance of self monitoring

Phone + Group Weight Loss Intervention

Contact number	Contact type[a]	Topics
1	Face-to-face	Welcome to program; program goals; weight loss mechanism; DASH eating plan
2	Phone	Physical Activity: Exercising safely, benefits of exercise, and barriers to exercise
3	Face-to-face	Calorie reduction; healthy breakfast; fruit & veggie intake; **PA session #1** of 3
4	Phone	Physical Activity: Stretching, warming up, strength training, and finding time to exercise
5	Phone	Healthy Eating: Understanding the difference between serving size and portion size and counting calories
6	Phone	Managing Time: Prioritizing and planning for healthy activities
7	Face-to-face	Healthy lunch; smart shopping and quick meals; environmental triggers for PA; **PA session #2** of 3; **Personal Feedback Report #1 of 2**
8	Phone	Stress Management: Stress and relaxation
9	Phone	Healthy Eating: Altering food preparation methods and eating patterns for dinner
10	Phone	Healthy Eating: Snacking patterns, triggers for snacking, and strategies for healthier snacking
11	Face-to-face	Dining out (making healthy choices); ;**PA session #3** of 3;
12	Phone	Support Systems: making use of family support and finding other support systems
13	Phone	Negative and positive thoughts; identifying negative thoughts, turning negative thoughts into positive ones, and using positive affirmation
14	Phone	Relapse prevention; special occasions; (TBD)
15	Face-to-face	Celebration of successes; Relapse prevention and weight maintenance: successful strategies for long-term weight loss; **Personal Feedback Report #2 of 2**

[a] Blue shading indicates face-to-face session

Section 3 Phase III. Maintenance of Weight Loss Intervention (Months 13-24)

Heart Healthy Lenoir participants were eligible to take part in the Weight Loss Maintenance (WLM) phase of the program if they lost ≥ 8 lbs. during the weight loss phase of the program.

The weight loss maintenance program lasted approximately 1 year and was conducted via telephone only. Participants were randomized into one of two treatment groups. Group 1 received a total of 36 phone coaching calls. The calls occurred weekly for the first 6 months (24 sessions over 24 weeks) and then bi-weekly for the last 6 months (12 sessions over 24 weeks). Group 2 received a total of 18 phone coaching calls. The calls occurred bi-weekly for the first 6 months (12 sessions over 24 weeks) and then monthly for the last 6 months (6 sessions over 24 weeks). Participants were encouraged to find a specified day and time to complete the call (e.g. every Monday at 2:00 PM) to assist with the consistency of the frequency of the calls. However, if a session had to be moved (planned or unplanned) the protocols that follow were used: weekly calls must be at least 3 days apart, bi-weekly calls must be at least 7 days apart and monthly calls must be at least 10 days apart.

The core structure of the HHL WLM program was based on the "traffic light" model developed by Wing.[1] With this system, if the participant had either lost weight or gained ≤ 2.99 lbs. they were considered to be in the "green zone." If a participant was in the green zone they were given positive reinforcement in the form of verbal praise and encouraged to continue their healthy weight loss maintenance behaviors. If the participant had gained between 3.0 and 4.99 lbs. they were considered to be in the "yellow zone." If a participant was in the yellow zone the counselor focused on problem solving skills to help the participant get their weight back down to the green zone. Finally, if a participant had gained five or more pounds they were considered to be in the red zone. Participants in the red zone where counseled to resume active weight loss.

The phone coach was a trained masters-level registered dietitian. During each phone session the following information would be collected from the participant: current body weight, days of self-monitoring since the last session (for weight and physical activity). At the end of each session the coach would encourage the participant to set personal goals for the next session.

REFERENCE

1. Wing RR, Tate DF, Gorin AA, Raynor HA, Fava JL. A self-regulation program for maintenance of weight loss. *N Engl J Med.* 2006;355(15):1563-1571.
