# Supplementary material for: A community-based lifestyle and weight loss intervention promoting a Mediterranean-style diet pattern evaluated in the stroke belt of North Carolina: the Heart Healthy Lenoir Project
Source: BMC Public Health. 2016 Aug 5;16:732. doi: 10.1186/s12889-016-3370-9 (PMC4975883; doi:10.1186/s12889-016-3370-9)
Supplement: Additional file 2: — BRFSS Survey Results for Overweight and Obesity: Eastern North Carolina and North Carolina. (DOCX 14 kb) [file 12889_2016_3370_MOESM2_ESM.docx]

Supplementary Table 1 BRFSS[^1^](#_ENREF_1) Survey Results for Overweight and Obesity: Eastern North Carolina and North Carolina

| Year | Total  Responding | Overweight  BMI 25 to 29.9 | | | Obese  BMI ≥ 30 | | |
| --- | --- | --- | --- | --- | --- | --- | --- |
|  | n | n | % | 95% CI | n | % | 95% CI |
| North Carolina--All | | | | | | | |
| 2011 | 10,790 | 3,944 | 36.0 | 34.5-37.5 | 2,981 | 29.1 | 27.7-30.6 |
| 2012 | 11,113 | 4,012 | 36.2 | 35.1-37.4 | 3,369 | 29.6 | 28.5-30.7 |
| 2013 | 8,296 | 2,973 | 36.7 | 35.3-38.2 | 2,550 | 29.4 | 28.1-30.7 |
| 2014 | 6,621 | 2,396 | 35.9 | 34.5-37.3 | 1,979 | 29.7 | 28.4-31.0 |
| North Carolina--Eastern | | | | | | | |
| 2011 | 2,915 | 1,078 | 36.0 | 33.2-39.0 | 927 | 32.4 | 29.7-35.2 |
| 2012 | 3,681 | 1,344 | 36.1 | 34.1-38.1 | 1,202 | 31.7 | 29.8-33.7 |
| 2013 | 2,527 | 890 | 36.9 | 34.3-39.7 | 881 | 32.4 | 29.9-35.0 |
| 2014 | 2,077 | 754 | 36.6 | 34.0-39.3 | 688 | 32.0 | 29.6-34.4 |

*Abbreviations:* BRFSS, behavioral risk factor surveillance system; BMI, body mass index

REFERENCE

1. North Carolina Department of Health and Human Services Division of Public Health, State Center for Health Statistics. Annual Survey Results: Behavioral Risk Factor Surveillance System (BRFSS). [<http://www.schs.state.nc.us/data/brfss/survey.htm>]. Accessed 07 Apr 2016.
