# Supplementary material for: A community-based lifestyle and weight loss intervention promoting a Mediterranean-style diet pattern evaluated in the stroke belt of North Carolina: the Heart Healthy Lenoir Project
Source: BMC Public Health. 2016 Aug 5;16:732. doi: 10.1186/s12889-016-3370-9 (PMC4975883; doi:10.1186/s12889-016-3370-9)
Supplement: Additional file 3: — BRFSS Survey Results for Exercise: Eastern North Carolina and North Carolina. (DOCX 15 kb) [file 12889_2016_3370_MOESM3_ESM.docx]

Supplementary Table 2 BRFSS[^1^](#_ENREF_1) Survey Results for Exercise: Eastern North Carolina and North Carolina

| Year | Total  Responding | Participate in Physical Activity or Exercise^a^ | | | | | |
| --- | --- | --- | --- | --- | --- | --- | --- |
|  |  | Yes | | | No | | |
|  | n | n | % | 95% CI | n | % | 95% CI |
| North Carolina--All | | | | | | | |
| 2011 | 11,130 | 8,053 | 73.3 | 71.9-74.6 | 3,077 | 26.7 | 25.4-28.1 |
| 2012 | 11,889 | 8,641 | 75.1 | 74.1-76.1 | 3,248 | 24.9 | 23.9-25.9 |
| 2013 | 8,405 | 5,993 | 73.4 | 72.1-74.7 | 2,412 | 26.6 | 25.3-27.9 |
| 2014 | 7,281 | 5,493 | 76.8 | 75.6-77.9 | 1,788 | 23.2 | 22.1-24.4 |
| North Carolina--Eastern | | | | | | | |
| 2011 | 3,029 | 2,100 | 69.8 | 67.0-72.5 | 929 | 30.2 | 27.5-33.0 |
| 2012 | 3,918 | 2,789 | 74.2 | 72.4-76.0 | 1,129 | 25.8 | 24.0-27.6 |
| 2013 | 2,534 | 1,752 | 71.8 | 69.2-74.3 | 782 | 28.2 | 25.7-30.8 |
| 2014 | 2,270 | 1,664 | 74.1 | 71.8-76.2 | 606 | 25.9 | 23.8-28.2 |

*Abbreviations:* BRFSS, behavioral risk factor surveillance system

### ^a^ Exercise is categorized as your or no based on the following question: During the past month, other than your regular job, did you participate in any physical activities or exercises such as running, calisthenics, golf, gardening, or walking for exercise?

REFERENCE

1. North Carolina Department of Health and Human Services Division of Public Health, State Center for Health Statistics. Annual Survey Results: Behavioral Risk Factor Surveillance System (BRFSS). [<http://www.schs.state.nc.us/data/brfss/survey.htm>]. Accessed 07 Apr 2016.
